# Association Between Recreational Physical Activity and mTOR Signaling Pathway Protein Expression in Breast Tumor Tissue

**DOI:** 10.1158/2767-9764.CRC-22-0405

**Published:** 2023-03-07

**Authors:** Ting-Yuan David Cheng, Runzhi Zhang, Zhihong Gong, Bo Qin, Rikki A. Cannioto, Susmita Datta, Weizhou Zhang, Angela R. Omilian, Song Yao, Thaer Khoury, Chi-Chen Hong, Elisa V. Bandera, Christine B. Ambrosone

**Affiliations:** 1 Division of Cancer Prevention and Control, Department of Internal Medicine, The Ohio State University, Columbus, Ohio.; 2 Department of Biostatistics, University of Florida, Gainesville, Florida.; 3 Department of Cancer Prevention and Control, Roswell Park Comprehensive Cancer Center, Buffalo, New York.; 4 Cancer Epidemiology and Health Outcomes, Rutgers Cancer Institute of New Jersey, The State University of New Jersey, New Brunswick, New Jersey.; 5 Department of Pathology, Immunology and Laboratory Medicine, University of Florida, Gainesville, Florida.; 6 Department of Pathology & Laboratory Medicine, Roswell Park Comprehensive Cancer Center, Buffalo, New York.

## Abstract

Physical activity (PA) is associated with decreased signaling in the mTOR pathway in animal models of mammary cancer, which may indicate favorable outcomes. We examined the association between PA and protein expression in the mTOR signaling pathway in breast tumor tissue. Data on 739 patients with breast cancer, among which 125 patients had adjacent-normal tissue, with tumor expression for mTOR, phosphorylated (p)-mTOR, p-AKT, and p-P70S6K were analyzed. Self-reported recreational PA levels during the year prior to diagnosis were classified using the Centers for Disease Control and Prevention guideline as sufficient (for moderate or vigorous) PA or insufficient PA (any PA but not meeting the guideline) or no PA. We performed linear models for mTOR protein and two-part gamma hurdle models for phosphorylated proteins. Overall, 34.8% of women reported sufficient PA; 14.2%, insufficient PA; 51.0%, no PA. Sufficient (vs. no) PA was associated with higher expression for p-P70S6K [35.8% increase; 95% confidence interval (CI), 2.6–80.2] and total phosphoprotein (28.5% increase; 95% CI, 5.8–56.3) among tumors with positive expression. In analyses stratified by PA intensity, sufficient versus no vigorous PA was also associated with higher expression levels of mTOR (beta = 17.7; 95% CI, 1.1–34.3) and total phosphoprotein (28.6% higher; 95% CI, 1.4–65.0 among women with positive expression) in tumors. The study found that guideline-concordant PA levels were associated with increased mTOR signaling pathway activity in breast tumors. Studying PA in relation to mTOR signaling in humans may need to consider the complexity of the behavioral and biological factors.

**Significance:**

PA increases energy expenditure and limits energy utilization in the cell, which can influence the mTOR pathway that is central to sensing energy influx and regulating cell growth. We studied exercise-mediated mTOR pathway activities in breast tumor and adjacent-normal tissue. Despite the discrepancies between animal and human data and the limitations of our approach, the findings provide a foundation to study the mechanisms of PA and their clinical implications.

## Introduction

Physical activity (PA) is associated with lower breast cancer risk among women and associated with lower risk of recurrence and mortality among patients with breast cancer (1–3). Given that the epidemiologic evidence regarding PA is well documented, mechanisms, including decreasing levels of sex hormone, insulin, glucose, leptin, inflammation, and adiposity, as well as improving immune function, are being hypothesized as potential pathways in the association between PA and improved survival rates among people with cancer (1). Although there are emerging data showing changes in blood biomarkers associated with PA among patients with cancer or survivors (4–10), data on changes in tumor markers due to PA largely found no significant exercise-mediated effects (11, 12). Randomized trials of exercise did not observe changes in Ki-67, an important marker for cell proliferation in breast cancer (13, 14). The number of participants was small (≤100) in these trials and observational studies examining tumor tissue. Thus, studies on potential pathways in breast tumor tissue or tumor microenvironments are needed to improve our understanding of the direct impact of PA on patients with cancer. Evidence that PA is associated with tumor markers would provide insights into developing precision, mechanism-based interventions (15), such as promoting exercise among patients with specific tumor markers to provide a larger benefit of PA in these patients.

A mechanism of PA for reducing body weight is increasing energy expenditure as well as limiting the availability of energy utilized in the cell. The mTOR signaling pathway is a sensor of energy influx and plays a key role in regulating protein synthesis, cell growth, and cell survival (16). Overactivation of the mTOR signaling pathway due to obesity, a result of positive energy balance, is associated with poor outcomes in patients with breast cancer (17, 18). Data from animal models have shown that PA decreases the protein expression levels of activated mTOR, AKT, and phosphorylated P70S6K (p-P70S6K)—important factors in the mTOR signaling pathway—in mammary carcinomas (19, 20). However, the relationship between physical activity and mTOR activities in the tumors of patients with breast cancer has not been reported.

Here, we investigated the association between recreational PA levels and mTOR pathway activation as measured by a panel of protein and phosphoprotein expression levels in tumors from participants enrolled in the Women's Circle of Health Study (WCHS). Previous analysis of a consortium of Black women including WCHS showed vigorous PA is associated with a lower risk of breast cancer (21). We hypothesized that women who had “sufficient” recreational PA levels would have lower mTOR pathway activity in breast tumors compared with women who had “insufficient” or no PA.

## Materials and Methods

### Study Participants

Study participants were women with breast cancer recruited between 2001 and 2015 for the WCHS, a multisite case–control study conducted in New York City and 10 counties in eastern New Jersey. Details on study recruitment have been described elsewhere (22, 23). The design of the case–control study recruited Black women and White women in 1:1 ratio; in a later stage, the tissue collection effort was more focused on the enrolled Black women than White women. All participants provided written informed consent. The protocol was approved by all relevant Institutional Review Boards and conducted in accordance with the Declaration of Helsinki. In brief, the cases included patients who self-identified as Black women or as White women between 20 and 75 years of age, able to speak English, with no previous history of cancer other than nonmelanoma skin cancer and who were within 9 months of having received a diagnosis of primary, histologically confirmed, invasive breast cancer or carcinoma *in situ*. Of patients eligible for inclusion, >95% allowed for the use of their tumor tissue as part of the informed consent form. Clinical and tumor characteristics were obtained from pathology reports. Formalin-fixed paraffin-embedded tissue specimens were used for tissue microarray (TMA) construction that included at least two tumor tissue cores and an adjacent-normal tissue core when available per patient. In total, samples from 865 cases included in TMAs were available for laboratory assays. After immunostaining, tumor tissue cores <25 cells for scoring were excluded. Subsequently, 739 cases (668 invasive breast cancer and 71 carcinoma *in situ*) who had at least one tumor tissue core scored with any of the mTOR pathway proteins assayed and data on PA variables were retained for statistical analyses. A subset of cases (*N* = 125) who had adjacent-normal tissue paired with the tumor tissue were also analyzed.

### IHC and Image Analysis

TMAs consisting of both tumor and adjacent-normal tissue cores were sectioned at 5 μm and stained by IHC methods for mTOR (clone 7C10), phosphorylated mTOR (p-mTOR, Ser2448), phosphorylated AKT (p-AKT, Ser473), and p-P70S6K (T389). Detailed methods for staining are given elsewhere (24). Stained slides were digitally imaged at a magnification of × 20 using the Aperio ScanScope XT (Leica Biosystems) digital slide scanner system, and images were manually annotated to identify tumors for analysis. Automated image analysis was performed on the annotated regions using validated algorithms with minor adjustments for cell shape and intensity thresholds. Specific locations (cytoplasm for mTOR and p-mTOR expression, and both cytoplasm and nuclei for p-AKT and p-P70S6K) were scored for staining intensity (0, none; 1+, partial or weak; 2+, moderate; or 3+, strong) and for the percentage of positive cells in each category. A histologic score (H-score) at the core level was calculated by the formula [1 × (% cells 1+) + 2 × (% cells 2+) + 3 × (% cells 3+)] × 100 (25). The core-level data were collapsed into case-level data using a cellularity-weighted approach (26). In addition, the p-mTOR/mTOR ratio, defined as the H-score of p-mTOR divided by the H-score of mTOR, and total phosphoprotein, derived as the summation of H-scores from p-mTOR, p-AKT, and p-P70S6K, were also examined.

### PA and Covariate Measurements

PA levels during the year prior to diagnosis were obtained via a structured home interview. During the period of participant recruitment, two versions of PA questionnaires were used. In WCHS 1 (2002–2012), participants reported exercise, sport, and leisure-time activities they had participated in for ≥1 hour per week for at least 3 months, the average duration (hours per week), and the age of engagement. A metabolic equivalent of task (MET) value was then assigned to each reported activity according to the Compendium of Physical Activity (27, 28). In WCHS 2 (2012–2017), participants reported weekly duration of moderate PA (MET 2.5 to <6) and vigorous PA (MET ≥6) during the year before diagnosis (29). We combined the data collected from the two versions of questionnaires and classified individuals as having *sufficient* (150 minutes/week for moderate PA or 75 minutes/week for vigorous PA), insufficient (any PA but not meeting the level of sufficient PA), or no PA, based on the aerobic component of the 2008 Physical Activity Guidelines for Americans by the Centers for Disease Control and Prevention (CDC; ref. 30).

Information on age, race, educational level, menopausal status, and medication use was obtained during the in-person interviews. A food frequency questionnaire was self-administered for usual dietary intake during the year before diagnosis (31). Anthropometric measurements were obtained by trained staff using a standardized protocol described elsewhere (32). Standing height was measured to the nearest 0.1 cm; weight was measured using a Tanita TBF-300A scale. Body mass index (BMI) was calculated as weight in kilograms divided by height in meters squared (33). Clinical and tumor characteristics, including the expression status of hormone receptors [HR; i.e., estrogen receptors (ER) and progesterone receptors] and HER2, were obtained from pathology reports.

### Statistical Analysis

We assessed protein expression levels in tumor and adjacent-normal tissue according to the three PA levels using one-way ANOVA. Regression models were only performed for protein expression in tumor tissue because the number of patients with the adjacent-normal tissue would not provide sufficient statistical power. Linear regression was performed to estimate the difference in H-scores for mTOR between the categories of PA levels because it was normally distributed. For the phosphoproteins, because a high proportion of tumors were negative for expression (H-score = 0 for 12% of p-mTOR staining, 27% of p-AKT, and 21% of p-P70S6K; Supplementary Table S1), gamma hurdle models were used to model the positive (nonzero) versus negative (zero) data with a logistic model and then the positive data with a gamma model with a log-link. ORs and percentage differences were converted from the regression coefficients of the respective parts of the gamma hurdle models. The covariates included age (continuous), race (Black or White), educational level (≤high school, some college, or ≥college graduate), menopausal status (premenopausal or postmenopausal), BMI (continuous), history of diabetes (yes or no; defined as using any oral or injection medication for diabetes), breast cancer molecular subtype (HR^+^/HER2^−^, HR^+^/HER2^+^, HR^−^/HER2^+^, or HR^−^/HER2^−^), tumor grade (low, intermediate, or high), tumor size (<1.0, 1.0–1.9, or ≥2.0 cm), and disease stage (American Joint Committee on Cancer staging system stage 0/I, II, or III/IV). Because BMI and history of diabetes can be intermediate variables on the causal pathway between PA and mTOR signaling, we also performed models without each of these two variables as a sensitivity analysis. Because dietary caloric intake is associated with PA levels and can modulate p-mTOR and p-AKT activities (34), total energy intake was additionally adjusted in a sensitivity analysis. Furthermore, to examine specific PA intensity in association with mTOR signaling pathway activity, we estimated the associations for (i) moderate PA (3 to <6 METs of exercise for at least 150 minutes/week defined as sufficient) among patients without vigorous PA and (ii) vigorous PA (≥6 METs of exercise for at least 75 minutes/week defined as sufficient) regardless the level of moderate PA. Exploratory stratification analyses were performed by race, BMI, menopausal status, history of diabetes, total energy intake levels, breast cancer stage, tumor grade, ER status, tumor size, and lymph node status because these variables were associated with energy balance or the mTOR pathway signaling. All tests of statistical significance were two sided; a *P* value less than 0.05 was considered statistically significant. All analyses were *a priori*, and the results were not adjusted for multiplicity.

### Data Availability

The data supporting the findings of this study are not publicly available to protect patient privacy. The data will be made available to authorized researchers with the approval of the WCHS committee and relevant Institutional Review Boards.

### Ethics Approval and Consent to Participate

All participants provided written informed consent and a release for access to medical records, pathology data, and tumor tissues prior to study participation. The protocol was approved by the Institutional Review Boards of all participating institutions, including Roswell Park Comprehensive Cancer Center and Rutgers Cancer Institute of New Jersey.

## Results

Table 1 gives the characteristics of participants overall and according to PA levels based on the CDC Guideline. Eighty percent of participants were Black women and 20% were White women. Overall, 14.2% and 34.8% of women reported insufficient and sufficient PA during the year before diagnosis, respectively. Approximately half (51.0%) of women reported no regular PA. White women (47.3%) were more likely than Black women (31.6%) to report sufficient PA. Premenopausal women (vs. postmenopausal), with lower (vs. higher) BMI, and without (vs. with) history of diabetes were more likely to meet the CDC guideline for PA. PA levels did not differ by the clinical and tumor characteristics.

**TABLE 1:** Characteristics of breast cancer cases from the WCHS according to physical activity level

		Physical activity levels			
	All cases	No	Insufficient	Sufficient	*P* value, *X*^2^ tests
*Characteristic*	*n* (column %)	*n* (row %)	*n* (row %)	*n* (row %)	
**All participants**	739 (100.0)	377 (51.0)	105 (14.2)	257 (34.8)	
**Race**					0.001
Black	591 (80.0)	319 (54.0)	85 (14.4)	187 (31.6)	
White	148 (20.0)	58 (39.2)	20 (13.5)	70 (47.3)	
**Age (years)**					0.007
<40	80 (10.8)	41 (51.2)	7 (8.8)	32 (40.0)	
40–49	212 (28.7)	99 (46.7)	26 (12.3)	87 (41.0)	
50–59	232 (31.4)	112 (48.3)	34 (14.7)	86 (37.1)	
≥60	215 (29.1)	125 (58.1)	38 (17.7)	52 (24.2)	
**Educational level attainment**					<0.001
≤High school	317 (42.9)	187 (59.0)	46 (14.5)	84 (26.5)	
Some college	187 (25.3)	94 (50.3)	22 (11.8)	71 (38.0)	
≥College graduate	235 (31.8)	96 (40.9)	37 (15.7)	102 (43.4)	
**Menopausal status**					0.011
Premenopausal	334 (45.2)	164 (49.1)	37 (11.1)	133 (39.8)	
Postmenopausal	405 (54.8)	213 (52.6)	68 (16.8)	124 (30.6)	
**BMI**					0.001
<25.0	159 (21.5)	63 (39.6)	22 (13.8)	74 (46.5)	
25.0–29.9	205 (27.7)	102 (49.8)	27 (13.2)	76 (37.1)	
30.0–34.9	191 (25.8)	96 (50.3)	32 (16.8)	63 (33.0)	
≥35.0	183 (24.8)	115 (62.8)	24 (13.1)	44 (24.0)	
Missing	1 (0.1)	1 (100)	0 (0)	0 (0)	
**History of diabetes**					0.007
Never	632 (85.5)	313 (49.5)	85 (13.4)	234 (37.0)	
Ever	107 (14.5)	64 (59.8)	20 (18.7)	23 (21.5)	
**Tumor grade**					0.73
Low	103 (13.9)	50 (48.5)	12 (11.7)	41 (39.8)	
Intermediate	232 (31.4)	124 (53.4)	30 (12.9)	78 (33.6)	
High	311 (42.1)	166 (53.4)	44 (14.1)	101 (32.5)	
Missing	93 (12.6)	37 (39.8)	19 (20.4)	37 (39.8)	
**Tumor size (cm)**					0.30
<1.0	93 (12.6)	41 (44.1)	17 (18.3)	35 (37.6)	
1.0–1.9	248 (33.6)	127 (51.2)	30 (12.1)	91 (36.7)	
≥2.0	328 (44.4)	180 (54.9)	43 (13.1)	105 (32.0)	
Missing	70 (9.5)	29 (41.4)	15 (21.4)	26 (37.1)	
**AJCC stage**					0.39
0/I	350 (47.4)	167 (47.7)	54 (15.4)	129 (36.9)	
II	280 (37.9)	154 (55.0)	35 (12.5)	91 (32.5)	
III, IV	98 (13.3)	54 (55.1)	14 (14.3)	30 (30.6)	
Missing	11 (1.5)	2 (18.2)	2 (18.2)	7 (63.6)	
**Lymph node status**					0.45
Negative	373 (50.5)	182 (48.8)	59 (15.8)	132 (35.4)	
Positive	253 (34.2)	134 (53.0)	32 (12.6)	87 (34.4)	
Missing	113 (15.3)	61 (54.0)	14 (12.4)	38 (33.6)	
**Molecular subtype**					0.84
HR^+^/HER2^−^	437 (59.1)	230 (52.6)	56 (12.8)	151 (34.6)	
HR^+^/HER2^+^	90 (12.2)	43 (47.8)	16 (17.8)	31 (34.4)	
HR^−^/HER2^+^	40 (5.4)	19 (47.5)	6 (15.0)	15 (37.5)	
HR^−^/HER2^−^	120 (16.2)	64 (53.3)	13 (10.8)	43 (35.8)	
Missing	52 (7.0)	21 (40.4)	14 (26.9)	17 (32.7)	
**Invasiveness**					0.06
No	71 (9.6)	29 (40.8)	16 (22.5)	26 (36.6)	
Yes	668 (90.4)	348 (52.1)	89 (13.3)	231 (34.6)	
**PA intensity**					NA
Moderate PA	739 (100)	433 (58.6)	111 (15)	195 (26.4)	
Vigorous PA	739 (100)	599 (81.1)	30 (4.1)	110 (14.9)	

Abbreviations: AJCC, American Joint Committee on Cancer; BMI, body mass index calculated as weight in kilograms divided by height in meters squared; HR, hormone receptor; NA, not applicable; WCHS, Women's Circle of Health Study.

Examination of the mTOR pathway protein expression by PA levels (Table 2) suggested that women with sufficient versus no PA had significantly higher expression of p-AKT, p-P70S6K, and total phosphoprotein in tumors (all *P* < 0.05). Comparing protein expression in a subset of tumors with paired adjacent-normal tissue (Supplementary Table S2), there was an indication that mTOR pathway proteins had higher expression, that is, higher proportions of positive (H-score >0) expression and higher mean and median H-scores among those with positive expression, in tumors than in adjacent-normal tissue. Women with sufficient PA compared with those with no PA had higher mean and median protein expression levels in the adjacent-normal tissue although the proportions of positive expression were generally lower in the sufficient PA group (Supplementary Table S3).

**TABLE 2:** Physical activity levels and protein expression levels in the mTOR signaling pathway in tumor tissue

		Physical activity levels			
		No	Insufficient	Sufficient	*P* value,
Protein	No.	Median (IQR)	Median (IQR)	Median (IQR)	ANOVA
mTOR	720	138.34 (75.6–194.0)	140.5 (93.9–195.4)	141.2 (87.3–205.3)	0.29
p-mTOR	717	20.3 (1.3–87.1)	36.6 (5.3–89.9)	33.3 (2.7–82.0)	0.33
p-AKT	722	10.2 (0–58.0)	19.1 (0.3–92.3)	19.1 (0.2–79.8)	0.010
p-P70S6K	721	4.4 (0–66.7)	27.0 (1.3–144.5)	26.1 (0.7–115.5)	<0.001
Total phosphoprotein	705	94.6 (16.8–211.1)	172.0 (57.2–282.0)	129.5 (46.4–256.3)	<0.001
p-mTOR/mTOR	705	0.15 (0.02–0.59)	0.27 (0.07–0.54)	0.23 (0.03–0.60)	0.27

Abbreviations: ANOVA, analysis of variance; IQR, interquartile range.

In the regression analyses adjusting for covariates (Table 3), there was no association between PA and mTOR expression. However, sufficient PA versus no PA was associated with p-P70S6K expression (OR = 1.60; 95% CI, 1.00–2.59, positive vs. negative protein expression) with a borderline significance. Among women with positive expression of p-P70S6K, sufficient PA was associated with 35.8% (95% CI, 2.6–80.2) higher expression in breast tumor tissue. In addition, sufficient (vs. no) PA was associated with 28.5% (95% CI, 5.8–56.3) higher expression of total phosphoproteins among women with positive expression. After additionally adjusting for total energy intake (Supplementary Table S4), the associations for p-P70S6K and total phosphoprotein remained unchanged. We also performed models without two intermediate variables—BMI and history of diabetes (Supplementary Tables S5 and S6). The analysis showed that the association of sufficient PA with p-P70S6K and total phosphoprotein expression in breast tumors was essentially the same as the main model that included both variables.

**TABLE 3:** Associations between physical activity levels and protein expression levels in the mTOR signaling pathway

	Logistic model^b^					Gamma or linear model^c^				
Protein expression (Outcome)^a^	No.	Insufficient PA vs. no PA OR (95% CI)	*P*	Sufficient PA vs. no PA OR (95% CI)	*P*	No.	Insufficient PA vs. no PA Beta (95% CI)	*P*	Sufficient PA vs. no PA Beta (95% CI)	*P*
mTOR	NA	NA		NA		599	0.48 (−17.29 to 18.25)	0.96	9.01 (−3.96 to 21.98)	0.17
p-mTOR	593	1.53 (0.69–3.77)	0.32	1.55 (0.87–2.86)	0.14	523	9.0 (−20.6 to 52.3)	0.59	8.0 (−14.0 to 36.1)	0.51
p-AKT	598	1.62 (0.9–3.01)	0.11	1.36 (0.9–2.07)	0.14	421	10.8 (−22.0 to 60.6)	0.57	12.7 (−14.1 to 48.4)	0.38
p-P70S6K	595	1.33 (0.73–2.55)	0.37	1.60 (1.00–2.59)	0.052	467	9.4 (−24.6 to 62.1)	0.63	35.8 (2.6–80.2)	0.027
Total phosphoprotein	585	NA	NA	1.55 (0.56–4.77)	0.42	566	18.1 (−8.8 to 54.8)	0.21	28.5 (5.8–56.3)	0.010
p-mTOR/mTOR	587	1.48 (0.67–3.65)	0.36	1.75 (0.95–3.33)	0.08	490	13.9 (−16.0 to 57.3)	0.41	4.5 (−16.4 to 31.1)	0.70

Abbreviations: CI, confidence interval; NA, not applicable.

^a^All models adjusted for age, race, educational level, menopausal status, body mass index, diabetes history, molecular subtype, tumor grade, tumor size, and breast cancer stage.

^b^The first part of the gamma hurdle model, that is, modeling positive (H-score >0) versus negative (H-score = 0) expression with a logistic model.

^c^The second part of the gamma hurdle model, that is, modeling the positive expression (H-score >0) with a gamma model. mTOR was modeled in linear model.

The exploratory stratified analysis results are shown in Supplementary Tables S7–S16. Sufficient PA was associated with total phosphoprotein among Black women only (30.8% increase, *P* = 0.019). When stratified by biological factors, the sufficient PA-phosphoprotein expression association was more apparent in women with overweight or normal weight (vs. those with obesity), being menopause (vs. premenopausal), without diabetes (vs. with diabetes), with higher (vs. lower) total energy intake, and having earlier [0/I; vs. more advanced (II–IV)] stage of breast cancer, smaller (vs. larger) tumors in size, ER^+^ (vs. ER^−^) tumors, and lymph node-negative (vs. lymph node-positive) disease.

In the analysis by PA intensity, sufficient versus no moderate PA (Table 4) was not significantly associated with the protein expression levels. However, sufficient versus no vigorous PA (Table 5) was associated with higher expression of mTOR (beta = 17.7; 95% CI, 1.1–34.3, all women). There was a nonlinear association that insufficient versus no vigorous PA was associated with 57.0% higher (95% CI, 4.1–148.7, gamma model) and sufficient versus no vigorous PA was associated 28.6% higher (95% CI, 1.4–65.0, gamma model) total phosphoprotein expression.

**TABLE 4:** Associations between moderate physical activity levels and protein expression levels in the mTOR signaling pathway among patients with no vigorous PA

	Logistic model^b^					Gamma or linear model^c^				
Protein expression (Outcome)^a^	No.	Insufficient PA vs. no PA OR (95% CI)	*P*	Sufficient PA vs. no PA OR (95% CI)	*P*	No.	Insufficient PA vs. no PA Beta (95% CI)	*P*	Sufficient PA vs. no PA Beta (95% CI)	*P*
mTOR	NA	NA		NA		486	−0.17 (−20.07 to 19.72)	0.99	0.42 (−15.44 to 16.28)	0.96
p-mTOR	481	1.44 (0.62–3.8)	0.42	1.51 (0.75–3.26)	0.26	421	2.1 (−28.1 to 48.5)	0.91	1.8 (−23.1 to 36.2)	0.9
p-AKT	485	1.4 (0.75–2.74)	0.31	1.34 (0.81–2.26)	0.26	336	13.4 (−22.9 to 71.3)	0.53	6.1 (−23.1 to 48.2)	0.71
p-P70S6K	483	1.35 (0.69–2.8)	0.4	1.35 (0.78–2.43)	0.29	371	−12.4 (−42.3 to 37.3)	0.53	33.9 (−5.7 to 92.5)	0.091
Total phosphoprotein	477	NA		1.87 (0.5–9.38)	0.39	461	7.2 (−19.1 to 44.3)	0.64	20.8 (−4.6 to 54.1)	0.11
p-mTOR/mTOR	477	1.42 (0.61–3.76)	0.45	1.82 (0.86–4.17)	0.13	391	−0.3 (−28.7 to 42.6)	0.99	−2.5 (−26 to 29.8)	0.86

Abbreviations: CI, confidence interval; NA, not applicable.

^a^All models adjusted for age, race, education, menopausal status, body mass index, diabetes history, molecular subtype, tumor grade, tumor size, breast cancer stage.

^b^The first part of the gamma hurdle model, that is, modeling positive (H-score >0) versus negative (H-score = 0) expression with a logistic model.

^c^The second part of the gamma hurdle model, that is, modeling the positive expression (H-score >0) with a gamma model. mTOR was modeled in linear model.

**TABLE 5:** Associations between *vigorous* physical activity levels and expression levels of proteins in the mTOR signaling pathway.

	Logistic model^b^					Gamma or linear model^c^				
Protein expression (Outcome)^a^	No.	Insufficient PA vs. no PA OR (95% CI)	*P*	Sufficient PA vs. no PA OR (95% CI)	*P*	No.	Insufficient PA vs. no PA Beta (95% CI)	*P*	Sufficient PA vs. no PA Beta (95% CI)	*P*
mTOR	NA	NA		NA		599	8.1 (−20.4 to 36.7)	0.58	17.7 (1.1–34.3)	0.040
p-mTOR	593	1.46 (0.39–9.51)	0.63	1.38 (0.65–3.29)	0.44	523	20.1 (−27 to 111.3)	0.48	10.1 (−16.9 to 48.3)	0.51
p-AKT	598	2.45 (0.87–8.78)	0.12	1.15 (0.68–1.99)	0.61	421	42.2 (−15.7 to 158.7)	0.20	11.5 (−19.3 to 57.3)	0.52
p-P70S6K	595	1.34 (0.48–4.77)	0.61	1.81 (0.96–3.69)	0.08	467	61.4 (−7.1 to 206.7)	0.11	26.7 (−9.3 to 80.4)	0.16
Total phosphoprotein	585	NA	NA	0.89 (0.26–4.18)	0.87	566	57.0 (4.1–148.7)	0.040	28.6 (1.4 to 65.0)	0.041
p-mTOR/mTOR	587	1.33 (0.35–8.7)	0.72	1.46 (0.66–3.7)	0.39	490	39.9 (−13.1 to 141.1)	0.19	7.9 (−17.9 to 44.1)	0.59

Abbreviations: CI, confidence interval; NA, not applicable.

^a^All models adjusted for age, race, educational level, menopausal status, body mass index, diabetes history, molecular subtype, tumor grade, tumor size, and breast cancer stage.

^b^The first part of the gamma hurdle model, that is, modeling positive (H-score >0) versus negative (H-score = 0) expression with a logistic model.

^c^The second part of the gamma hurdle model, that is, modeling the positive expression (H-score >0) with a gamma model. mTOR was modeled in linear model.

## Discussion

In this study population of women with newly diagnosed breast cancer, sufficient PA that met the aerobic portion of the CDC guideline recommendation a year before the diagnosis was associated with higher levels of p-P70S6K and total phosphoprotein expression in breast tumor tissue. The associations were also observed for vigorous PA, but not for moderate PA among patients who did not report any vigorous PA.

PA involves a broad range of dimensions, including frequency, intensity, duration, purpose (long-term training vs. acute exercise), and type (aerobic, resistance, and flexibility). In general, PA reduces circulating growth factors and hormones that can trigger the mTOR signaling pathway. However, little is known about the extent to which those PA dimensions are related to the mTOR signaling pathway in human tumor tissue. Resistance training leads to increased mTOR activation, with mTOR being a key protein for increasing muscle size and strength (35–37). In rats, the activity of P70S6K in skeleton muscle increases 6 hours after exercise and is tightly associated with changes in muscle mass after 6 weeks of training (36). A similar observation has also been made in humans, with p-P70S6K and p-mTOR expression increasing after resistance training in training-accustomed young healthy men (37). No other type of PA (besides resistance training) has been reported to activate the mTOR signaling pathway in muscle. Interestingly, preclinical evidence suggests that mTOR activation by resistance PA is not through classical growth factor/PI3K/Akt signaling but likely through the activation of tuberous sclerosis complex 2, an mTOR regulator (38). This mechanism may explain why PA reduces circulating growth factors, but in our study, PA was associated with higher mTOR activation in breast tumors. Because our study did not examine mTOR expression in skeleton muscle, a study examining mTOR in both skeletal and tumor tissue would provide important data on whether changes associated with PA for mTOR signaling in tumors are similar to those in muscle.

The findings of our study were inconsistent with those of preclinical studies focusing on breast cancer. Jiang and colleagues compared the effects of PA (low-intensity, consistent, self-determined wheel running that mimicked the national recommendation of 10,000 steps), restricted energy intake, and no PA on tumor incidence and mTOR pathway activation in tumors using a well-designed mouse model (19). The study found that the mammary gland cancer incidence rate was lower in the PA group than in the sedentary group. In mammary tumors, levels of p-Akt (Ser473), p-P70S6K (Thr398), and p-4E-BP1 (Thr37/46) detected by Western blotting were downregulated in the PA group as well as in the mildly restricted energy group albeit to a lesser extent, both compared with sedentary controls. Those findings support the hypothesis that mTOR may be a mechanism through which PA is associated with reduced breast cancer risk. However, our study showed the opposite result, that is, PA was associated with higher mTOR signaling pathway activity in breast tumors. The mechanism underlying our finding is unclear, but our results suggested that how PA influences mTOR pathway activation in human breast cancer may be more complex than in preclinical models. For example, the animal model controlled energy intake in the PA group to be the same as the sedentary controls, resulting in a “negative” energy balance (92% of the controls) and weight reduction in the PA group (19). In our study, however, we were unable to control participants’ energy intake, and women with a high level of PA might have higher energy intake than those with a lower level of PA. Also, the validity of energy intake assessment from a single food frequency questionnaire is limited. Although our analysis adjusting total energy intake using regression methods did not materially change our findings, the adjustment may not be sufficient to reduce the influence of energy intake on our findings. In addition, total energy intake in humans varies widely between individuals, and cellular amino acids, glucose, and ATP/AMP concentrations from food can promote mTOR activation. We also observed relatively low mTOR pathway activities among the non-PA group. The group had a sedentary lifestyle and a higher proportion of being obese, an indication of a positive energy balance. However, the observation is inconsistent with our previous finding that higher versus lower BMI was associated with higher mTOR activities in breast tumors (24). In that study, however, PA levels were not considered. Thus, it is important to confirm whether PA has a dominant effect compared with BMI or total energy intake on the mTOR pathway activities, as our modeling results showed that these two variables had limited contribution to the main effect of PA. Also, studying how mTOR signaling factors, such as insulin-like growth factors (IGF), affect the pathway activities in tumors among patients with a sedentary versus active lifestyle is needed. Our findings highlight the challenge of comparing exposures related to energy intake and expenditure in animal models with human observational studies.

Epidemiologic evidence assessing PA levels and tumor mTOR and IGF pathway-related changes is not consistent across different cancer types and tissue markers. Among patients with colorectal cancer, there was no association between PA assessed after diagnosis and insulin receptor substrate 1, a mediator of insulin and IGF (39). In a prospective study that assessed PA at baseline before cancer diagnosis, higher (vs. lower) levels of nonoccupational PA were associated with lower expression levels of proteins involved in the Warburg effect, that is, upregulation of aerobic glycolysis in cancer cells signaled by PI3K/Akt, in rectal tumors among women (40). However, the association was opposite for colon cancer among women and rectal cancer among men. For breast cancer, in a preoperative exercise intervention trial to increase PA to 200 minutes of exercise per week, including 40 minutes of strength training and 180 minutes of moderate-intensity aerobic exercise, the intervention did not affect tumor gene expression (13). However, in a randomized trial of 32 overweight/obese patients with breast cancer, expression of PI3Kinase genes, upstream signaling of mTOR, significantly increased after aerobic exercise plus caloric restriction (14). More data are needed to conclude whether different types, doses, and schedules of PA can affect IGF or mTOR signaling in tumors.

Our results from adjacent-normal tissue and stratified analyses provide additional support for the main finding. Among the adjacent-normal tissue, we also observed higher mTOR pathway activities among participants with sufficient PA compared with no PA. However, the interpretation is limited by the fact that not all cases had analyzable adjacent-normal tissue despite our best effort to collect and assay these TMA cores. Normal breast tissue from healthy women without breast cancer would provide ideal samples for testing our hypothesis. The stratified analysis results are plausible because the PA-mTOR association was more apparent among participants with less influence from obesity and diabetes, factors that can affect the mTOR pathway activities. Also, previous research has shown that mTOR activities may be higher among patients with breast cancer with earlier (vs. more advanced) stage, lower (vs. higher) grade, smaller (vs. larger) tumors, and ER^+^ (vs. ER^−^) tumors (24). These clinical and pathologic factors should be considered when studying and interpreting the hypothesized associations.

Other limitations of our study include potential reversed causality, recall error from self-report, and confounding. The information on prediagnostic PA was collected after the breast cancer diagnosis, and women may have changed their PA levels due to preclinical symptoms although the questionnaire inquired about PA levels in the year before cancer diagnosis. Our questionnaire may not have covered all types and modes of PA, such as resistance or muscle-strengthening exercise as well as nonrecreational activities, and the accuracy of self-report PA is likely affected by recall errors. A prospective study with objective measures of PA is warranted to confirm the findings. Our analysis according to the moderate or vigorous PA levels might have been influenced by each other because of the high degree of overlap among individuals engaging in both levels of PA. For example, among those who reported any amount of vigorous PA, 60.0% reported moderate PA, and this can be a reason that we observed a higher tumor total phosphoprotein expression among the insufficient versus no vigorous PA group. Also, we are unable to adjust all possible confounders. For example, women with a higher PA level may be more engaged in screening behavior (i.e., receiving a mammogram) than women with lower PA levels or no PA. Adjusting for tumor size and stage in our analysis may have reduced confounding due to screening because screening is more likely to identify smaller tumors and earlier stages of disease compared with nonscreening-detected breast cancer. In addition, the number of proteins assessed in this study may not be large enough. It may be necessary to measure a larger number of proteins in assessing mTOR signaling pathway activity to further understand associations with PA.

It is important to note that patients with breast cancer and breast cancer survivors should engage in any PA and increase their activity levels (41). Exercise during breast cancer treatment may provide benefits, such as improving quality of life and managing adverse treatment effects (42). There is clear evidence that PA assessed before as well as after diagnosis is associated with reduced risk of mortality in women with long-term disease-free living or stable breast cancer (41, 43).

In conclusion, we found indications of higher mTOR signaling pathway activity in breast tumor tissue among women with sufficient PA levels compared with women with no PA. These findings are inconsistent with evidence from animal models indicating that exercise may be associated with lower mTOR signaling pathway activity in mammary cancer. Validation is needed in future research that uses a prospective study design and a more objective assessment of PA and considers the complexity of behavioral and biological variations of energy balance.

## Supplementary Material

Supplemental Table 1 Supplemental Table 1 shows the distribution of protein expression in H-score

Supplemental Table 2 Supplemental Table 2 shows protein expression in breast cancer tissue and paired adjacent normal tissue.

Supplemental Table 3 Supplemental Table 3 shows protein expression distribution in adjacent normal tissue by PA level.

Supplemental Table 4 Supplemental Table 4 shows the associations adjusting covariates and total energy intake.

Supplemental Table 5 Supplemental Table 5 reports the association without adjusting BMI as a sensitivity analysis.

Supplemental Table 6 Supplemental Table 6 reports the association without adjusting diabetes history as a sensitivity analysis.

Supplemental Table 7 Supplemental Table 7 reported stratified analysis for Black and White race.

Supplemental Table 8 Supplemental Table 8 reported stratified analysis for obesity and normal weight/overweight.

Supplemental Table 9 Supplemental Table 9 reported stratified analysis for postmenopausal and premenopausal women.

Supplemental Table 10 Supplemental Table 10 reported stratified analysis for women with and without history of diabetes.

Supplemental Table 11 Supplemental Table 11 reported stratified analysis for breast cancer stage 0/I and stage II/III/IV.

Supplemental Table 12 Supplemental Table 12 reported stratified analysis for low/intermediate-grade tumors and high-grade tumors.

Supplemental Table 13 Supplemental Table 13 reported stratified analysis for tumors <2cm and ≥2 cm in diameter.

Supplemental Table 14 Supplemental Table 14 reported stratified analysis for ER+ tumors and ER- tumors.

Supplemental Table 15 Supplemental Table 15 reported stratified analysis for lymph node positive status and lymph node negative status.

Supplemental Table 16 Supplemental Table 16 reported stratified analysis for high (above the median) and low (at or below the median) total energy intake levels.
