# Supplementary material for: Association Between Recreational Physical Activity and mTOR Signaling Pathway Protein Expression in Breast Tumor Tissue
Source: Cancer Res Commun. 2023 Mar 7;3(3):395–403. doi: 10.1158/2767-9764.CRC-22-0405 (PMC9990525; doi:10.1158/2767-9764.CRC-22-0405)
Supplement: Supplemental Table 1 — shows the distribution of protein expression in H-score [file crc-22-0405-s01.docx]

Supplemental Table 1. Distribution of protein expression by H-score

| **Protein** | **No.** | **% Negative (H-score = 0)** | **Positive expression (H-score > 0)** | | | |
| --- | --- | --- | --- | --- | --- | --- |
|  |  |  | **Mean** | **SD** | **Median** | **Interquartile range** |
| mTOR | 720 | 1% | 139.8 | 74.1 | 139.8 | 83.6 - 198.2 |
| p-mTOR | 717 | 12% | 57.8 | 60.8 | 37.4 | 6.6 - 91.7 |
| p-AKT | 722 | 27% | 65.4 | 72.9 | 36.9 | 9.2 - 103.9 |
| p-p70S6K | 721 | 21% | 74.8 | 88.1 | 40.8 | 2.9 - 117.2 |
| Total phosphoprotein | 705 | 3% | 163.4 | 153.7 | 126.1 | 32.2 - 252.3 |
| p-mTOR/mTOR | 705 | 11% | 1.87 | 25.48 | 0.27 | 0.07 - 0.64 |
