# Supplementary material for: Association Between Recreational Physical Activity and mTOR Signaling Pathway Protein Expression in Breast Tumor Tissue
Source: Cancer Res Commun. 2023 Mar 7;3(3):395–403. doi: 10.1158/2767-9764.CRC-22-0405 (PMC9990525; doi:10.1158/2767-9764.CRC-22-0405)
Supplement: Supplemental Table 2 — shows protein expression in breast cancer tissue and paired adjacent normal tissue. [file crc-22-0405-s02.docx]

Supplemental Table 2. Protein expression distribution in breast tumor tissue and their paired adjacent normal tissue

| **Protein** | **N** | **% negative (H-score = 0)** | **Positive expression (H-score > 0)** | | | |
| --- | --- | --- | --- | --- | --- | --- |
| **Tumor tissue** |  |  | **Mean** | **SD** | **Median** | **Inter-quartile range** |
| mTOR | 121 | 1% | 119.4 | 71.6 | 116.0 | 58.7 - 164.2 |
| p-mTOR | 119 | 18% | 53.8 | 61.0 | 27.4 | 3.3 - 84.4 |
| p-AKT | 125 | 38% | 55.7 | 72.8 | 20.2 | 5.1 - 98.4 |
| p-p70S6K | 125 | 22% | 59.6 | 75.2 | 26.6 | 1.8 - 98.7 |
| Total phosphoprotein | 101 | 6% | 125.4 | 129.2 | 97.9 | 15.1 - 188.6 |
| Normalized p-mTOR | 100 | 18% | 0.54 | 0.79 | 0.22 | 0.03 - 0.85 |
| **Adjacent Normal tissue** | |  |  |  |  |  |
| mTOR | 121 | 8% | 103.8 | 65.1 | 100.0 | 50.6 - 145.2 |
| p-mTOR | 119 | 35% | 45.5 | 47.4 | 26.8 | 7.1 - 61.4 |
| p-AKT | 125 | 46% | 49.1 | 57.2 | 19.9 | 6.6 - 87.5 |
| p-p70S6K | 125 | 47% | 61.5 | 59.4 | 43.3 | 12.5 - 98.0 |
| Total phosphoprotein | 101 | 19% | 109.0 | 118.5 | 62.5 | 15.9 - 161.7 |
| Normalized p-mTOR | 100 | 32% | 0.64 | 1.27 | 0.27 | 0.14 - 0.66 |
