# Supplementary material for: Association Between Recreational Physical Activity and mTOR Signaling Pathway Protein Expression in Breast Tumor Tissue
Source: Cancer Res Commun. 2023 Mar 7;3(3):395–403. doi: 10.1158/2767-9764.CRC-22-0405 (PMC9990525; doi:10.1158/2767-9764.CRC-22-0405)
Supplement: Supplemental Table 3 — shows protein expression distribution in adjacent normal tissue by PA level. [file crc-22-0405-s03.docx]

Supplemental Table 3. Protein expression distribution in adjacent normal tissue by PA level

| **Protein** | **N** | **% negative (H-score = 0)** | **Positive expression (H-score > 0)** | | | |
| --- | --- | --- | --- | --- | --- | --- |
| **No PA** | | | **Mean** | **SD** | **Median** | **Inter-quartile range** |
| mTOR | 59 | 7% | 96.0 | 66.7 | 86.0 | 39.1 - 148.5 |
| p-mTOR | 58 | 28% | 42.2 | 46.8 | 24.8 | 6.1 - 56.3 |
| p-AKT | 60 | 42% | 46.0 | 53.4 | 19.9 | 6.9 - 78.1 |
| p-p70S6K | 58 | 50% | 47.3 | 51.5 | 26.2 | 11.1 - 62.1 |
| Total phosphoprotein | 48 | 15% | 91.6 | 104.1 | 44.9 | 12.5 - 149.5 |
| Normalized p-mTOR | 48 | 25% | 0.80 | 1.68 | 0.26 | 0.15 - 0.66 |
| **Insufficient PA** | | |  |  |  |  |
| mTOR | 13 | 8% | 106.2 | 53.1 | 96.9 | 68.7 - 139.4 |
| p-mTOR | 16 | 50% | 49.5 | 56.4 | 28.0 | 14.0 - 62.8 |
| p-AKT | 14 | 57% | 51.8 | 58.5 | 30.8 | 8.2 - 82.1 |
| p-p70S6K | 16 | 50% | 61.4 | 64.8 | 43.6 | 18.6 - 79.5 |
| Total phosphoprotein | 13 | 23% | 115.0 | 148.6 | 71.9 | 22.0 - 131.8 |
| Normalized p-mTOR | 13 | 46% | 0.50 | 0.51 | 0.30 | 0.18 - 0.66 |
| **Sufficient PA** | | |  |  |  |  |
| mTOR | 49 | 10% | 112.8 | 66.2 | 109.5 | 59.6 - 145.8 |
| p-mTOR | 45 | 40% | 49.5 | 47.1 | 41.7 | 9.6 - 61.9 |
| p-AKT | 51 | 47% | 52.6 | 63.3 | 17.9 | 7.4 - 102.3 |
| p-p70S6K | 51 | 43% | 75.6 | 63.9 | 65.1 | 15.2 - 105.8 |
| Total phosphoprotein | 40 | 22% | 130.0 | 126.3 | 73.5 | 22.0 - 226.5 |
| Normalized p-mTOR | 39 | 36% | 0.46 | 0.43 | 0.36 | 0.13 - 0.58 |
