# Supplementary material for: Association Between Recreational Physical Activity and mTOR Signaling Pathway Protein Expression in Breast Tumor Tissue
Source: Cancer Res Commun. 2023 Mar 7;3(3):395–403. doi: 10.1158/2767-9764.CRC-22-0405 (PMC9990525; doi:10.1158/2767-9764.CRC-22-0405)
Supplement: Supplemental Table 4 — shows the associations adjusting covariates and total energy intake. [file crc-22-0405-s04.docx]

Supplemental Table 4. Associations between physical activity levels and mTOR signaling pathway protein expression levels, additionally adjust for *total energy intake* among women who had complete food frequency questionnaire data

|  |  | Physical activity levels | | | | |
| --- | --- | --- | --- | --- | --- | --- |
| Protein expression (Outcome)^a^ | No. | No | Insufficient |  | Sufficient |  |
|  |  |  | Difference or odds ratio (95% CI) | P value | Difference or odds ratio (95% CI) | P value |
| **mTOR** |  |  |  |  |  |  |
| Linear model | 589 | Ref. | 0.41 (-17.48 - 18.30) | 0.96 | 7.74 (-5.25 - 20.74) | 0.24 |
| **p-mTOR** |  |  |  |  |  |  |
| Logistic model^b^ | 583 | Ref. | 1.7 (0.74 - 4.43) | 0.24 | 1.52 (0.84 - 2.8) | 0.17 |
| Gamma model^c^ | 515 | Ref. | 6.2% (-23.1% - 49.3%) | 0.72 | 6.9% (-15.4% - 35.3%) | 0.58 |
| **p-AKT** |  |  |  |  |  |  |
| Logistic model^b^ | 588 | Ref. | 1.5 (0.83 - 2.8) | 0.19 | 1.35 (0.89 - 2.06) | 0.16 |
| Gamma model^c^ | 416 | Ref. | 14.5% (-19.7% - 66.6%) | 0.46 | 10.8% (-15.6% - 45.9%) | 0.45 |
| **p-P70S6K** |  |  |  |  |  |  |
| Logistic model^b^ | 585 | Ref. | 1.34 (0.72 - 2.61) | 0.38 | 1.51 (0.95 - 2.46) | 0.08 |
| Gamma model^c^ | 461 | Ref. | 10.6% (-24% - 64.5%) | 0.59 | 37.4% (3.6% - 82.6%) | 0.022 |
| **Total phosphoprotein** |  |  |  |  |  |  |
| Logistic model^b^ | 575 | Ref. | NA | NA | 1.34 (0.48 - 4.18) | 0.59 |
| Gamma model^c^ | 557 | Ref. | 18.3% (-9.1% - 55.8%) | 0.21 | 27.6% (4.8% - 55.6%) | 0.013 |
| **p-mTOR/mTOR** |  |  |  |  |  |  |
| Logistic model^b^ | 577 | Ref. | 1.65 (0.72 - 4.32) | 0.27 | 1.71 (0.93 - 3.26) | 0.09 |
| Gamma model^c^ | 482 | Ref. | 10.5% (-19.1% - 53.6%) | 0.53 | 3.4% (-17.9% - 30.5%) | 0.78 |

^a^All models adjusted for age, race, educational level, menopausal status, body mass index, diabetes history, molecular subtype, tumor grade, tumor size, breast cancer stage, and total energy intake.

^b^The first part of the gamma hurdle model, i.e., modeling positive (H-score >0) vs. negative (H-score =0) expression with a logistic model.

^c^The second part of the gamma hurdle model, i.e., modeling the positive expression (H-score >0) with a gamma model.

Abbreviations: CI, confidence interval; NA, not applicable; Ref., reference.
