# Supplementary material for: Association Between Recreational Physical Activity and mTOR Signaling Pathway Protein Expression in Breast Tumor Tissue
Source: Cancer Res Commun. 2023 Mar 7;3(3):395–403. doi: 10.1158/2767-9764.CRC-22-0405 (PMC9990525; doi:10.1158/2767-9764.CRC-22-0405)
Supplement: Supplemental Table 5 — reports the association without adjusting BMI as a sensitivity analysis. [file crc-22-0405-s05.docx]

Supplemental Table 5. Sensitivity analysis - models without adjusting BMI

|  |  | Physical activity levels | | | | |
| --- | --- | --- | --- | --- | --- | --- |
| Protein expression (Outcome)^a^ | No. | No | Insufficient |  | Sufficient |  |
|  |  |  | Difference or odds ratio (95% CI) | P value | Difference or odds ratio (95% CI) | P value |
| **mTOR** |  |  |  |  |  |  |
| Linear model | 599 | Ref. | 0.15 (-17.57 - 17.87) | 0.99 | 8.44 (-4.36 - 21.24) | 0.2 |
| **p-mTOR** |  |  |  |  |  |  |
| Logistic model^b^ | 594 | Ref. | 1.54 (0.7 - 3.78) | 0.31 | 1.52 (0.86 - 2.79) | 0.16 |
| Gamma model^c^ | 523 | Ref. | 6.6% (-21.9% - 48%) | 0.69 | 4.5% (-16.4% - 30.9%) | 0.7 |
| **p-AKT** |  |  |  |  |  |  |
| Logistic model^b^ | 599 | Ref. | 1.61 (0.9 - 2.99) | 0.12 | 1.36 (0.9 - 2.05) | 0.15 |
| Gamma model^c^ | 422 | Ref. | 9.9% (-22.4% - 58.7%) | 0.6 | 12.4% (-13.7% - 47%) | 0.38 |
| **p-P70S6K** |  |  |  |  |  |  |
| Logistic model^b^ | 596 | Ref. | 1.32 (0.72 - 2.53) | 0.38 | 1.58 (1 - 2.55) | 0.054 |
| Gamma model^c^ | 468 | Ref. | 8.3% (-25.2% - 60.3%) | 0.67 | 33.7% (1.7% - 76.3%) | 0.031 |
| **Total phosphoprotein** |  |  |  |  |  |  |
| Logistic model^b^ | 586 | Ref. | NA | NA | 1.42 (0.52 - 4.35) | 0.51 |
| Gamma model^c^ | 567 | Ref. | 16.1% (-10.2% - 51.9%) | 0.26 | 25.2% (3.6% - 51.7%) | 0.019 |
| **p-mTOR/mTOR** |  |  |  |  |  |  |
| Logistic model^b^ | 587 | Ref. | 1.45 (0.65 - 3.58) | 0.39 | 1.67 (0.91 - 3.16) | 0.11 |
| Gamma model^c^ | 490 | Ref. | 14.7% (-15.3% - 58%) | 0.38 | 1.5% (-18.7% - 27.1%) | 0.89 |

^a^All models adjusted for age, race, educational level, menopausal status, diabetes history, molecular subtype, tumor grade, tumor size, and breast cancer stage.

^b^The first part of the gamma hurdle model, i.e., modeling positive (H-score >0) vs. negative (H-score =0) expression with a logistic model.

^c^The second part of the gamma hurdle model, i.e., modeling the positive expression (H-score >0) with a gamma model.

Abbreviations: CI, confidence interval; NA, not applicable; Ref., reference.
