# Supplementary material for: Association Between Recreational Physical Activity and mTOR Signaling Pathway Protein Expression in Breast Tumor Tissue
Source: Cancer Res Commun. 2023 Mar 7;3(3):395–403. doi: 10.1158/2767-9764.CRC-22-0405 (PMC9990525; doi:10.1158/2767-9764.CRC-22-0405)
Supplement: Supplemental Table 6 — reports the association without adjusting diabetes history as a sensitivity analysis. [file crc-22-0405-s06.docx]

|  |  | Physical activity levels | | | | |
| --- | --- | --- | --- | --- | --- | --- |
| Protein expression (Outcome)^a^ | No. | No | Insufficient |  | Sufficient |  |
|  |  |  | Difference or odds ratio (95% CI) | P value | Difference or odds ratio (95% CI) | P value |
| **mTOR** |  |  |  |  |  |  |
| Linear model | 599 | Ref. | 0.68 (-17.06 - 18.43) | 0.94 | 8.87 (-4.08 - 21.82) | 0.18 |
| **p-mTOR** |  |  |  |  |  |  |
| Logistic model^b^ | 593 | Ref. | 1.53 (0.69 - 3.75) | 0.32 | 1.56 (0.87 - 2.87) | 0.14 |
| Gamma model^c^ | 523 | Ref. | 10.7% (-19.2% - 54.5%) | 0.53 | 7.6% (-14.4% - 35.5%) | 0.53 |
| **p-AKT** |  |  |  |  |  |  |
| Logistic model^b^ | 598 | Ref. | 1.62 (0.91 - 3.01) | 0.11 | 1.36 (0.9 - 2.07) | 0.14 |
| Gamma model^c^ | 421 | Ref. | 9.9% (-22.6% - 59.3%) | 0.6 | 12.8% (-14.1% - 48.6%) | 0.37 |
| **p-P70S6K** |  |  |  |  |  |  |
| Logistic model^b^ | 595 | Ref. | 1.3 (0.71 - 2.5) | 0.41 | 1.61 (1.01 - 2.6) | 0.049 |
| Gamma model^c^ | 467 | Ref. | 10.3% (-24% - 63.6%) | 0.6 | 36.5% (3% - 81.3%) | 0.025 |
| **Total phosphoprotein** |  |  |  |  |  |  |
| Logistic model^b^ | 585 | Ref. | NA | NA | 1.5 (0.54 - 4.56) | 0.45 |
| Gamma model^c^ | 566 | Ref. | 17.9% (-8.9% - 54.6%) | 0.21 | 28.6% (5.9% - 56.4%) | 0.0097 |
| **p-mTOR/mTOR** |  |  |  |  |  |  |
| Logistic model^b^ | 587 | Ref. | 1.48 (0.66 - 3.65) | 0.37 | 1.75 (0.95 - 3.34) | 0.077 |
| Gamma model^c^ | 490 | Ref. | 14% (-15.9% - 57.3%) | 0.41 | 4.4% (-16.5% - 30.9%) | 0.7 |

Supplemental Table 6. Sensitivity analysis - models without adjusting history of diabetes

^a^All models adjusted for age, race, educational level, menopausal status, body mass index, molecular subtype, tumor grade, tumor size, and breast cancer stage.

^b^The first part of the gamma hurdle model, i.e., modeling positive (H-score >0) vs. negative (H-score =0) expression with a logistic model.

^c^The second part of the gamma hurdle model, i.e., modeling the positive expression (H-score >0) with a gamma model.

Abbreviations: CI, confidence interval; NA, not applicable; Ref., reference.
