# Supplementary material for: Association Between Recreational Physical Activity and mTOR Signaling Pathway Protein Expression in Breast Tumor Tissue
Source: Cancer Res Commun. 2023 Mar 7;3(3):395–403. doi: 10.1158/2767-9764.CRC-22-0405 (PMC9990525; doi:10.1158/2767-9764.CRC-22-0405)
Supplement: Supplemental Table 7 — reported stratified analysis for Black and White race. [file crc-22-0405-s07.docx]

Supplemental Table 7. Stratified analysis by race

1. **Black women**

|  |  | Physical activity levels | | | | | |
| --- | --- | --- | --- | --- | --- | --- | --- |
| Protein expression (Outcome)^a^ | No. | No | Insufficient |  | Sufficient |  |  |
|  |  |  | Difference or odds ratio (95% CI) | P value | Difference or odds ratio (95% CI) | P value |  |
| **mTOR** |  |  |  |  |  |  |  |
| Linear model | 471 | Ref. | -1.75 (-22.44 - 18.94) | 0.87 | 7.19 (-8.13 - 22.52) | 0.36 |  |
| **p-mTOR** |  |  |  |  |  |  |  |
| Logistic model^b^ | 466 | Ref. | 1.08 (0.46 - 2.87) | 0.87 | 2.08 (0.98 - 4.76) | 0.065 |  |
| Gamma model^c^ | 418 | Ref. | 26.9% (-11.2% - 85.7%) | 0.19 | 9.4% (-15.5% - 42.3%) | 0.5 |  |
| **p-AKT** |  |  |  |  |  |  |  |
| Logistic model^b^ | 469 | Ref. | 1.88 (0.96 - 3.87) | 0.074 | 1.41 (0.88 - 2.29) | 0.16 |  |
| Gamma model^c^ | 330 | Ref. | 13.9% (-23.8% - 74.8%) | 0.53 | 15.8% (-15.8% - 60.1%) | 0.36 |  |
| **p-P70S6K** |  |  |  |  |  |  |  |
| Logistic model^b^ | 465 | Ref. | 1.21 (0.61 - 2.51) | 0.6 | 1.48 (0.86 - 2.58) | 0.16 |  |
| Gamma model^c^ | 363 | Ref. | 5.6% (-30.4% - 64.5%) | 0.79 | 29.3% (-5.6% - 77.8%) | 0.096 |  |
| **Total phosphoprotein** |  |  |  |  |  |  |  |
| Logistic model^b^ | 461 | Ref. | NA | NA | 1.9 (0.57 - 7.88) | 0.33 |  |
| Gamma model^c^ | 446 | Ref. | 27% (-5.7% - 73.7%) | 0.12 | 30.8% (4.4% - 64.5%) | 0.019 |  |
| **p-mTOR/mTOR** |  |  |  |  |  |  |  |
| Logistic model^b^ | 463 | Ref. | 1.04 (0.44 - 2.79) | 0.93 | 2.29 (1.05 - 5.45) | 0.045 |  |
| Gamma model^c^ | 396 | Ref. | 34.1% (-3.6% - 90.8%) | 0.087 | 2.8% (-19.3% - 31.4%) | 0.83 |  |

^a^All models adjusted for the same covariates except for the stratified variable.

^b^The first part of the gamma hurdle model, i.e., modeling positive (H-score >0) vs. negative (H-score =0) expression with a logistic model.

^c^The second part of the gamma hurdle model, i.e., modeling the positive expression (H-score >0) with a gamma model.

Abbreviations: CI, confidence interval; NA, not applicable; Ref., reference

1. **White women**

|  |  | Physical activity levels | | | | |
| --- | --- | --- | --- | --- | --- | --- |
| Protein expression (Outcome)^a^ | No. | No | Insufficient |  | Sufficient |  |
|  |  |  | Difference or odds ratio (95% CI) | P value | Difference or odds ratio (95% CI) | P value |
| **mTOR** |  |  |  |  |  |  |
| Linear model | 128 | Ref. | 1.76 (-32.36 - 35.87) | 0.92 | 12.57 (-11.03 - 36.17) | 0.29 |
| **p-mTOR** |  |  |  |  |  |  |
| Logistic model^b^ | 127 | Ref. | 4.33 (0.59 - 91.02) | 0.21 | 1.24 (0.4 - 3.96) | 0.71 |
| Gamma model^c^ | 105 | Ref. | -45.6% (-74.2% - 44.3%) | 0.12 | -19.7% (-54.6% - 32.7%) | 0.43 |
| **p-AKT** |  |  |  |  |  |  |
| Logistic model^b^ | 129 | Ref. | 0.54 (0.12 - 2.39) | 0.41 | 0.79 (0.29 - 2.14) | 0.65 |
| Gamma model^c^ | 91 | Ref. | -45.1% (-81.6% - 67.1%) | 0.2 | -17.3% (-62.3% - 79.6%) | 0.56 |
| **p-P70S6K** |  |  |  |  |  |  |
| Logistic model^b^ | 130 | Ref. | 3.73 (0.65 - 28.98) | 0.16 | 2.95 (0.88 - 10.85) | 0.088 |
| Gamma model^c^ | 104 | Ref. | 18.8% (-54.5% - 210.4%) | 0.73 | 82.1% (-6.8% - 255.8%) | 0.083 |
| **Total phosphoprotein** |  |  |  |  |  |  |
| Logistic model^b^ | 124 | Ref. | NA | NA | 1.5 (0.01 - 160.55) | 0.85 |
| Gamma model^c^ | 120 | Ref. | -23% (-57.1% - 43.2%) | 0.37 | 3.3% (-32.3% - 57.8%) | 0.87 |
| **p-mTOR/mTOR** |  |  |  |  |  |  |
| Logistic model^b^ | 124 | Ref. | 5.31 (0.68 - 115.91) | 0.17 | 1.4 (0.43 - 4.83) | 0.58 |
| Gamma model^c^ | 97 | Ref. | -9.5% (-61.5% - 128.3%) | 0.79 | -16.8% (-53.5% - 47.6%) | 0.52 |

^a^All models adjusted for the same covariates except for the stratified variable.

^b^The first part of the gamma hurdle model, i.e., modeling positive (H-score >0) vs. negative (H-score =0) expression with a logistic model.

^c^The second part of the gamma hurdle model, i.e., modeling the positive expression (H-score >0) with a gamma model.

Abbreviations: CI, confidence interval; NA, not applicable; Ref., reference.
