# Supplementary material for: Association Between Recreational Physical Activity and mTOR Signaling Pathway Protein Expression in Breast Tumor Tissue
Source: Cancer Res Commun. 2023 Mar 7;3(3):395–403. doi: 10.1158/2767-9764.CRC-22-0405 (PMC9990525; doi:10.1158/2767-9764.CRC-22-0405)
Supplement: Supplemental Table 8 — reported stratified analysis for obesity and normal weight/overweight. [file crc-22-0405-s08.docx]

Supplemental Table 8. Stratified analysis by BMI

1. **Obesity (BMI ≥30 kg/m^2^)**

|  |  | Physical activity levels | | | | |
| --- | --- | --- | --- | --- | --- | --- |
| Protein expression (Outcome)^a^ | No. | No | Insufficient |  | Sufficient |  |
|  |  |  | Difference or odds ratio (95% CI) | P value | Difference or odds ratio (95% CI) | P value |
| **mTOR** |  |  |  |  |  |  |
| Linear model | 303 | Ref. | -8.75 (-33.07 - 15.57) | 0.48 | 2.69 (-16.4 - 21.79) | 0.78 |
| **p-mTOR** |  |  |  |  |  |  |
| Logistic model^b^ | 301 | Ref. | 1.44 (0.45 - 5.78) | 0.57 | 1.51 (0.57 - 4.45) | 0.43 |
| Gamma model^c^ | 269 | Ref. | 18.5% (-19.2% - 78.3%) | 0.39 | 25% (-7.4% - 70%) | 0.15 |
| **p-AKT** |  |  |  |  |  |  |
| Logistic model^b^ | 300 | Ref. | 1.47 (0.65 - 3.57) | 0.37 | 0.96 (0.51 - 1.81) | 0.9 |
| Gamma model^c^ | 212 | Ref. | 16.4% (-29.5% - 99.7%) | 0.55 | 13.7% (-24.5% - 73.3%) | 0.53 |
| **p-P70S6K** |  |  |  |  |  |  |
| Logistic model^b^ | 301 | Ref. | 0.94 (0.4 - 2.32) | 0.88 | 0.86 (0.44 - 1.73) | 0.67 |
| Gamma model^c^ | 234 | Ref. | 12.4% (-34.1% - 99.3%) | 0.66 | 24.8% (-19.5% - 95.4%) | 0.29 |
| **Total phosphoprotein** |  |  |  |  |  |  |
| Logistic model^b^ | 297 | Ref. | NA | NA | 0.24 (0.02 - 3.76) | 0.27 |
| Gamma model^c^ | 291 | Ref. | 23.6% (-13.7% - 80.9%) | 0.24 | 20.3% (-9.6% - 61.2%) | 0.2 |
| **p-mTOR/mTOR** |  |  |  |  |  |  |
| Logistic model^b^ | 300 | Ref. | 1.39 (0.42 - 5.63) | 0.61 | 1.5 (0.56 - 4.5) | 0.44 |
| Gamma model^c^ | 257 | Ref. | 31% (-11.2% - 98.7%) | 0.19 | 13% (-17.9% - 56.8%) | 0.45 |

^a^All models adjusted for the same covariates except for the stratified variable.

^b^The first part of the gamma hurdle model, i.e., modeling positive (H-score >0) vs. negative (H-score =0) expression with a logistic model.

^c^The second part of the gamma hurdle model, i.e., modeling the positive expression (H-score >0) with a gamma model.

Abbreviations: CI, confidence interval; NA, not applicable; Ref., reference.

1. **Normal weight/overweight (BMI <30 kg/m^2^)**

|  |  | Physical activity levels | | | | |
| --- | --- | --- | --- | --- | --- | --- |
| Protein expression (Outcome)^a^ | No. | No | Insufficient |  | Sufficient |  |
|  |  |  | Difference or odds ratio (95% CI) | P value | Difference or odds ratio (95% CI) | P value |
| **mTOR** |  |  |  |  |  |  |
| Linear model | 296 | Ref. | 13.49 (-13.42 - 40.4) | 0.32 | 13.86 (-4.27 - 31.99) | 0.13 |
| **p-mTOR** |  |  |  |  |  |  |
| Logistic model^b^ | 292 | Ref. | 1.69 (0.54 - 6.51) | 0.4 | 1.34 (0.62 - 2.97) | 0.45 |
| Gamma model^c^ | 254 | Ref. | -8% (-45.9% - 63%) | 0.75 | -9% (-36.8% - 31%) | 0.59 |
| **p-AKT** |  |  |  |  |  |  |
| Logistic model^b^ | 298 | Ref. | 2.09 (0.88 - 5.44) | 0.11 | 1.84 (1.04 - 3.3) | 0.037 |
| Gamma model^c^ | 209 | Ref. | -4.6% (-47.9% - 79.2%) | 0.87 | 7.7% (-28.1% - 61.3%) | 0.7 |
| **p-P70S6K** |  |  |  |  |  |  |
| Logistic model^b^ | 294 | Ref. | 2 (0.78 - 5.69) | 0.17 | 2.47 (1.27 - 4.99) | 0.0092 |
| Gamma model^c^ | 233 | Ref. | 31% (-24.7% - 137.3%) | 0.33 | 62.9% (9.4% - 142.5%) | 0.011 |
| **Total phosphoprotein** |  |  |  |  |  |  |
| Logistic model^b^ | 288 | Ref. | NA | NA | 1.83 (0.52 - 7.5) | 0.36 |
| Gamma model^c^ | 275 | Ref. | 21.4% (-18.2% - 84%) | 0.32 | 38.2% (5.3% - 81.6%) | 0.016 |
| **p-mTOR/mTOR** |  |  |  |  |  |  |
| Logistic model^b^ | 287 | Ref. | 1.66 (0.53 - 6.43) | 0.41 | 1.7 (0.76 - 3.99) | 0.2 |
| Gamma model^c^ | 233 | Ref. | -2.9% (-42.3% - 69.7%) | 0.91 | -12.6% (-38.3% - 23.9%) | 0.43 |

^a^All models adjusted for the same covariates except for the stratified variable.

^b^The first part of the gamma hurdle model, i.e., modeling positive (H-score >0) vs. negative (H-score =0) expression with a logistic model.

^c^The second part of the gamma hurdle model, i.e., modeling the positive expression (H-score >0) with a gamma model.

Abbreviations: CI, confidence interval; NA, not applicable; Ref., reference.
