# Supplementary material for: Association Between Recreational Physical Activity and mTOR Signaling Pathway Protein Expression in Breast Tumor Tissue
Source: Cancer Res Commun. 2023 Mar 7;3(3):395–403. doi: 10.1158/2767-9764.CRC-22-0405 (PMC9990525; doi:10.1158/2767-9764.CRC-22-0405)
Supplement: Supplemental Table 9 — reported stratified analysis for postmenopausal and premenopausal women. [file crc-22-0405-s09.docx]

Supplemental Table 9. Stratified analysis by menopausal status

1. **Postmenopausal women**

|  |  | Physical activity levels | | | | |
| --- | --- | --- | --- | --- | --- | --- |
| Protein expression (Outcome)^a^ | No. | No | Insufficient |  | Sufficient |  |
|  |  |  | Difference or odds ratio (95% CI) | P value | Difference or odds ratio (95% CI) | P value |
| **mTOR** |  |  |  |  |  |  |
| Linear model | 327 | Ref. | -8.44 (-30.56 - 13.67) | 0.45 | 9.48 (-8.61 - 27.56) | 0.3 |
| **p-mTOR** |  |  |  |  |  |  |
| Logistic model^b^ | 324 | Ref. | 1.59 (0.6 - 4.8) | 0.38 | 1.83 (0.81 - 4.4) | 0.16 |
| Gamma model^c^ | 283 | Ref. | 6.5% (-27.9% - 61.1%) | 0.76 | -7.8% (-33.3% - 28.5%) | 0.62 |
| **p-AKT** |  |  |  |  |  |  |
| Logistic model^b^ | 328 | Ref. | 1.4 (0.67 - 3.12) | 0.39 | 1.17 (0.63 - 2.2) | 0.62 |
| Gamma model^c^ | 242 | Ref. | 33.6% (-14% - 113.6%) | 0.21 | 28.9% (-12% - 90.4%) | 0.18 |
| **p-P70S6K** |  |  |  |  |  |  |
| Logistic model^b^ | 325 | Ref. | 1.39 (0.65 - 3.18) | 0.41 | 1.68 (0.87 - 3.34) | 0.13 |
| Gamma model^c^ | 250 | Ref. | 0.5% (-38.4% - 68.5%) | 0.98 | 87.6% (22% - 190.6%) | 0.0017 |
| **Total phosphoprotein** |  |  |  |  |  |  |
| Logistic model^b^ | 321 | Ref. | NA | NA | 0.89 (0.14 - 6.22) | 0.9 |
| Gamma model^c^ | 313 | Ref. | 17.5% (-14.3% - 63.5%) | 0.32 | 39.8% (6.4% - 84.4%) | 0.013 |
| **p-mTOR/mTOR** |  |  |  |  |  |  |
| Logistic model^b^ | 322 | Ref. | 1.57 (0.59 - 4.81) | 0.39 | 1.85 (0.81 - 4.49) | 0.16 |
| Gamma model^c^ | 266 | Ref. | 17.8% (-20.6% - 78.9%) | 0.41 | -7.3% (-32.9% - 28.9%) | 0.64 |

^a^All models adjusted for the same covariates except for the stratified variable.

^b^The first part of the gamma hurdle model, i.e., modeling positive (H-score >0) vs. negative (H-score =0) expression with a logistic model.

^c^The second part of the gamma hurdle model, i.e., modeling the positive expression (H-score >0) with a gamma model.

Abbreviations: CI, confidence interval; NA, not applicable; Ref., reference.

1. **Premenopausal women**

|  |  | Physical activity levels | | | | |
| --- | --- | --- | --- | --- | --- | --- |
| Protein expression (Outcome)^a^ | No. | No | Insufficient |  | Sufficient |  |
|  |  |  | Difference or odds ratio (95% CI) | P value | Difference or odds ratio (95% CI) | P value |
| **mTOR** |  |  |  |  |  |  |
| Linear model | 272 | Ref. | 17.92 (-12.77 - 48.61) | 0.25 | 8.22 (-11.04 - 27.48) | 0.4 |
| **p-mTOR** |  |  |  |  |  |  |
| Logistic model^b^ | 269 | Ref. | 1.42 (0.34 - 9.8) | 0.67 | 1.23 (0.51 - 3.07) | 0.65 |
| Gamma model^c^ | 240 | Ref. | 5% (-39.4% - 92.2%) | 0.86 | 35% (-4.6% - 91.6%) | 0.082 |
| **p-AKT** |  |  |  |  |  |  |
| Logistic model^b^ | 270 | Ref. | 2.12 (0.8 - 6.37) | 0.15 | 1.57 (0.87 - 2.84) | 0.13 |
| Gamma model^c^ | 179 | Ref. | -27.2% (-62.4% - 51.3%) | 0.34 | -5.6% (-39.8% - 48.6%) | 0.78 |
| **p-P70S6K** |  |  |  |  |  |  |
| Logistic model^b^ | 270 | Ref. | 1.25 (0.43 - 4.23) | 0.69 | 1.5 (0.75 - 3.11) | 0.26 |
| Gamma model^c^ | 217 | Ref. | 16.5% (-38.7% - 138.1%) | 0.63 | 2.4% (-32.7% - 56%) | 0.91 |
| **Total phosphoprotein** |  |  |  |  |  |  |
| Logistic model^b^ | 264 | Ref. | NA | NA | 1.84 (0.45 - 9.55) | 0.42 |
| Gamma model^c^ | 253 | Ref. | 15.7% (-26.6% - 89.9%) | 0.53 | 21.8% (-9.4% - 64.3%) | 0.17 |
| **p-mTOR/mTOR** |  |  |  |  |  |  |
| Logistic model^b^ | 265 | Ref. | 1.43 (0.34 - 10.03) | 0.66 | 1.68 (0.64 - 4.82) | 0.31 |
| Gamma model^c^ | 223 | Ref. | -5.4% (-42.9% - 65.2%) | 0.83 | 18.7% (-15.2% - 66.9%) | 0.31 |

^a^All models adjusted for the same covariates except for the stratified variable.

^b^The first part of the gamma hurdle model, i.e., modeling positive (H-score >0) vs. negative (H-score =0) expression with a logistic model.

^c^The second part of the gamma hurdle model, i.e., modeling the positive expression (H-score >0) with a gamma model.

Abbreviations: CI, confidence interval; NA, not applicable; Ref., reference.
