# Supplementary material for: Association Between Recreational Physical Activity and mTOR Signaling Pathway Protein Expression in Breast Tumor Tissue
Source: Cancer Res Commun. 2023 Mar 7;3(3):395–403. doi: 10.1158/2767-9764.CRC-22-0405 (PMC9990525; doi:10.1158/2767-9764.CRC-22-0405)
Supplement: Supplemental Table 10 — reported stratified analysis for women with and without history of diabetes. [file crc-22-0405-s10.docx]

Supplemental Table 10. Stratified analysis by history of diabetes

1. **Women with history of diabetes**

|  |  | Physical activity levels | | | | | |
| --- | --- | --- | --- | --- | --- | --- | --- |
| Protein expression (Outcome)^a^ | No. | No | Insufficient |  | Sufficient |  |  |
|  |  |  | Difference or odds ratio (95% CI) | P value | Difference or odds ratio (95% CI) | P value |  |
| **mTOR** |  |  |  |  |  |  |  |
| Linear model | 84 | Ref. | -23.52 (-72.61 - 25.57) | 0.34 | -7.03 (-53.73 - 39.67) | 0.76 |  |
| **p-mTOR** |  |  |  |  |  |  |  |
| Logistic model^b^ | 83 | Ref. | 0.48 (0.05 - 4.96) | 0.51 | 0.61 (0.06 - 8.05) | 0.68 |  |
| Gamma model^c^ | 73 | Ref. | 82.9% (-15.6% - 313.6%) | 0.14 | -30.4% (-67.7% - 58.7%) | 0.34 |  |
| **p-AKT** |  |  |  |  |  |  |  |
| Logistic model^b^ | 83 | Ref. | 1.68 (0.31 - 10.68) | 0.56 | 0.36 (0.07 - 1.75) | 0.21 |  |
| Gamma model^c^ | 61 | Ref. | 44.1% (-43.2% - 289.8%) | 0.43 | 203% (0.3% - 925.3%) | 0.022 |  |
| **p-P70S6K** |  |  |  |  |  |  |  |
| Logistic model^b^ | 83 | Ref. | 0.34 (0.07 - 1.55) | 0.17 | 1.13 (0.24 - 6.22) | 0.88 |  |
| Gamma model^c^ | 60 | Ref. | -67.8% (-88.9% - -5.99%) | 0.044 | 115.4% (-18.2% - 466.7%) | 0.13 |  |
| **Total phosphoprotein** |  |  |  |  |  |  |  |
| Logistic model^b^ | 82 | Ref. | NA | NA | NA | NA |  |
| Gamma model^c^ | 78 | Ref. | -3.5% (-48.7% - 85.6%) | 0.91 | 33.2% (-32.5% - 173.1%) | 0.37 |  |
| **p-mTOR/mTOR** |  |  |  |  |  |  |  |
| Logistic model^b^ | 82 | Ref. | 0.52 (0.06 - 5.23) | 0.55 | 0.71 (0.06 - 10.2) | 0.78 |  |
| Gamma model^c^ | 69 | Ref. | 63.5% (-26.6% - 289.1%) | 0.26 | -27.5% (-68.6% - 82.3%) | 0.43 |  |

^a^All models adjusted for the same covariates except for the stratified variable.

^b^The first part of the gamma hurdle model, i.e., modeling positive (H-score >0) vs. negative (H-score =0) expression with a logistic model.

^c^The second part of the gamma hurdle model, i.e., modeling the positive expression (H-score >0) with a gamma model.

Abbreviations: CI, confidence interval; NA, not applicable; Ref., reference.

1. **Women with no history of diabetes**

|  |  | Physical activity levels | | | | |
| --- | --- | --- | --- | --- | --- | --- |
| Protein expression (Outcome)^a^ | No. | No | Insufficient |  | Sufficient |  |
|  |  |  | Difference or odds ratio (95% CI) | P value | Difference or odds ratio (95% CI) | P value |
| **mTOR** |  |  |  |  |  |  |
| Linear model | 515 | Ref. | 4.48 (-15.22 - 24.18) | 0.66 | 11.62 (-2.24 - 25.48) | 0.1 |
| **p-mTOR** |  |  |  |  |  |  |
| Logistic model^b^ | 510 | Ref. | 1.79 (0.73 - 5.13) | 0.24 | 1.79 (0.95 - 3.47) | 0.075 |
| Gamma model^c^ | 450 | Ref. | -1.6% (-30.8% - 43.2%) | 0.93 | 5.6% (-17.3% - 35.1%) | 0.66 |
| **p-AKT** |  |  |  |  |  |  |
| Logistic model^b^ | 515 | Ref. | 1.62 (0.85 - 3.22) | 0.15 | 1.47 (0.95 - 2.3) | 0.087 |
| Gamma model^c^ | 360 | Ref. | 2.9% (-31.2% - 57.7%) | 0.89 | 6.3% (-21.2% - 43.9%) | 0.68 |
| **p-P70S6K** |  |  |  |  |  |  |
| Logistic model^b^ | 512 | Ref. | 1.76 (0.86 - 3.92) | 0.14 | 1.59 (0.96 - 2.67) | 0.073 |
| Gamma model^c^ | 407 | Ref. | 22.6% (-18.6% - 89.8%) | 0.33 | 37% (0.9% - 86.4%) | 0.035 |
| **Total phosphoprotein** |  |  |  |  |  |  |
| Logistic model^b^ | 503 | Ref. | NA | NA | 2.23 (0.67 - 9.03) | 0.22 |
| Gamma model^c^ | 488 | Ref. | 19.1% (-11.4% - 62.5%) | 0.25 | 24.1% (0.4% - 53.7%) | 0.041 |
| **p-mTOR/mTOR** |  |  |  |  |  |  |
| Logistic model^b^ | 505 | Ref. | 1.73 (0.66 - 4.57) | 0.27 | 1.93 (0.99 - 3.75) | 0.053 |
| Gamma model^c^ | 421 | Ref. | 12.4% (-20% - 61.3%) | 0.5 | 1.5% (-20% - 29%) | 0.91 |

^a^All models adjusted for the same covariates except for the stratified variable.

^b^The first part of the gamma hurdle model, i.e., modeling positive (H-score >0) vs. negative (H-score =0) expression with a logistic model.

^c^The second part of the gamma hurdle model, i.e., modeling the positive expression (H-score >0) with a gamma model.

Abbreviations: CI, confidence interval; NA, not applicable; Ref., reference.
