# Supplementary material for: Association Between Recreational Physical Activity and mTOR Signaling Pathway Protein Expression in Breast Tumor Tissue
Source: Cancer Res Commun. 2023 Mar 7;3(3):395–403. doi: 10.1158/2767-9764.CRC-22-0405 (PMC9990525; doi:10.1158/2767-9764.CRC-22-0405)
Supplement: Supplemental Table 11 — reported stratified analysis for breast cancer stage 0/I and stage II/III/IV. [file crc-22-0405-s11.docx]

Supplemental Table 11. Stratified analysis by breast cancer stage

1. **Stage 0/I**

|  |  | Physical activity levels | | | | |
| --- | --- | --- | --- | --- | --- | --- |
| Protein expression (Outcome)^a^ | No. | No | Insufficient |  | Sufficient |  |
|  |  |  | Difference or odds ratio (95% CI) | P value | Difference or odds ratio (95% CI) | P value |
| **mTOR** |  |  |  |  |  |  |
| Linear model | 265 | Ref. | 11.89 (-15.3 - 39.07) | 0.39 | 16.06 (-3.2 - 35.32) | 0.1 |
| **p-mTOR** |  |  |  |  |  |  |
| Logistic model^b^ | 260 | Ref. | 1.4 (0.39 - 6.09) | 0.63 | 3.21 (1.08 - 11.31) | 0.048 |
| Gamma model^c^ | 234 | Ref. | 74.5% (10.5% - 184%) | 0.013 | 30.4% (-3.8% - 77.3%) | 0.085 |
| **p-AKT** |  |  |  |  |  |  |
| Logistic model^b^ | 266 | Ref. | 3.11 (1.07 - 11.44) | 0.055 | 1.43 (0.74 - 2.83) | 0.29 |
| Gamma model^c^ | 204 | Ref. | -6% (-43.6% - 62.2%) | 0.81 | 44.1% (-2.6% - 114%) | 0.06 |
| **p-P70S6K** |  |  |  |  |  |  |
| Logistic model^b^ | 265 | Ref. | 1.47 (0.56 - 4.28) | 0.45 | 1.59 (0.77 - 3.34) | 0.21 |
| Gamma model^c^ | 206 | Ref. | 40.4% (-15.2% - 141.9%) | 0.19 | 62.5% (11.6% - 138.1%) | 0.01 |
| **Total phosphoprotein** |  |  |  |  |  |  |
| Logistic model^b^ | 259 | Ref. | NA | NA | 1.42 (0.28 - 8.31) | 0.68 |
| Gamma model^c^ | 251 | Ref. | 38.2% (-3.2% - 101.4%) | 0.077 | 49.1% (15.8% - 92.6%) | 0.0023 |
| **p-mTOR/mTOR** |  |  |  |  |  |  |
| Logistic model^b^ | 260 | Ref. | 1.4 (0.39 - 6.09) | 0.63 | 3.21 (1.08 - 11.31) | 0.048 |
| Gamma model^c^ | 220 | Ref. | 32.6% (-14.1% - 111.7%) | 0.2 | 28.9% (-4.6% - 74.8%) | 0.092 |

^a^All models adjusted for the same covariates except for the stratified variable.

^b^The first part of the gamma hurdle model, i.e., modeling positive (H-score >0) vs. negative (H-score =0) expression with a logistic model.

^c^The second part of the gamma hurdle model, i.e., modeling the positive expression (H-score >0) with a gamma model.

Abbreviations: CI, confidence interval; NA, not applicable; Ref., reference.

1. **Stage II-IV**

|  |  | Physical activity levels | | | | |
| --- | --- | --- | --- | --- | --- | --- |
| Protein expression (Outcome)^a^ | No. | No | Insufficient |  | Sufficient |  |
|  |  |  | Difference or odds ratio (95% CI) | P value | Difference or odds ratio (95% CI) | P value |
| **mTOR** |  |  |  |  |  |  |
| Linear model | 334 | Ref. | -10.44 (-34.06 - 13.17) | 0.38 | 2.74 (-14.99 - 20.46) | 0.76 |
| **p-mTOR** |  |  |  |  |  |  |
| Logistic model^b^ | 333 | Ref. | 1.43 (0.49 - 5.28) | 0.55 | 1.05 (0.5 - 2.24) | 0.9 |
| Gamma model^c^ | 289 | Ref. | -14.2% (-44.5% - 37.4%) | 0.5 | 8.3% (-23.6% - 54.9%) | 0.65 |
| **p-AKT** |  |  |  |  |  |  |
| Logistic model^b^ | 332 | Ref. | 1.03 (0.5 - 2.2) | 0.94 | 1.35 (0.78 - 2.36) | 0.28 |
| Gamma model^c^ | 217 | Ref. | -2.3% (-42.5% - 72.9%) | 0.93 | -7.3% (-38.4% - 40.5%) | 0.7 |
| **p-P70S6K** |  |  |  |  |  |  |
| Logistic model^b^ | 330 | Ref. | 1.05 (0.46 - 2.59) | 0.91 | 1.62 (0.83 - 3.28) | 0.17 |
| Gamma model^c^ | 261 | Ref. | -17.3% (-53.3% - 52.1%) | 0.49 | 13.2% (-27.6% - 78%) | 0.55 |
| **Total phosphoprotein** |  |  |  |  |  |  |
| Logistic model^b^ | 326 | Ref. | NA | NA | 2.04 (0.48 - 11.21) | 0.36 |
| Gamma model^c^ | 315 | Ref. | -3.7% (-34.4% - 44.8%) | 0.85 | 12.4% (-17.1% - 53.3%) | 0.43 |
| **p-mTOR/mTOR** |  |  |  |  |  |  |
| Logistic model^b^ | 327 | Ref. | 1.38 (0.46 - 5.15) | 0.59 | 1.27 (0.59 - 2.86) | 0.55 |
| Gamma model^c^ | 268 | Ref. | 14.8% (-24.5% - 80.3%) | 0.53 | -0.9% (-28.9% - 39.1%) | 0.96 |

^a^All models adjusted for the same covariates except for the stratified variable.

^b^The first part of the gamma hurdle model, i.e., modeling positive (H-score >0) vs. negative (H-score =0) expression with a logistic model.

^c^The second part of the gamma hurdle model, i.e., modeling the positive expression (H-score >0) with a gamma model.

Abbreviations: CI, confidence interval; NA, not applicable; Ref., reference.
