# Supplementary material for: Association Between Recreational Physical Activity and mTOR Signaling Pathway Protein Expression in Breast Tumor Tissue
Source: Cancer Res Commun. 2023 Mar 7;3(3):395–403. doi: 10.1158/2767-9764.CRC-22-0405 (PMC9990525; doi:10.1158/2767-9764.CRC-22-0405)
Supplement: Supplemental Table 12 — reported stratified analysis for low/intermediate-grade tumors and high-grade tumors. [file crc-22-0405-s12.docx]

Supplemental Table 12. Stratified analysis by tumor grade

1. **Low/intermediate grade**

|  |  | Physical activity levels | | | | |
| --- | --- | --- | --- | --- | --- | --- |
| Protein expression (Outcome)^a^ | No. | No | Insufficient |  | Sufficient |  |
|  |  |  | Difference or odds ratio (95% CI) | P value | Difference or odds ratio (95% CI) | P value |
| **mTOR** |  |  |  |  |  |  |
| Linear model | 312 | Ref. | 3.75 (-20.63 - 28.13) | 0.76 | 8.04 (-9.61 - 25.7) | 0.37 |
| **p-mTOR** |  |  |  |  |  |  |
| Logistic model^b^ | 311 | Ref. | 1.16 (0.36 - 4.62) | 0.82 | 1.55 (0.64 - 3.95) | 0.34 |
| Gamma model^c^ | 280 | Ref. | -0.6% (-29.1% - 41.8%) | 0.97 | 10.2% (-13.4% - 40.5%) | 0.43 |
| **p-AKT** |  |  |  |  |  |  |
| Logistic model^b^ | 314 | Ref. | 0.77 (0.34 - 1.79) | 0.52 | 1.06 (0.57 - 1.98) | 0.86 |
| Gamma model^c^ | 240 | Ref. | 7.2% (-31% - 72.6%) | 0.76 | 4.9% (-23.4% - 44.3%) | 0.77 |
| **p-P70S6K** |  |  |  |  |  |  |
| Logistic model^b^ | 313 | Ref. | 1.81 (0.77 - 4.7) | 0.19 | 1.58 (0.85 - 2.99) | 0.15 |
| Gamma model^c^ | 240 | Ref. | 5.7% (-34.8% - 77.6%) | 0.82 | 30.6% (-10% - 90.8%) | 0.14 |
| **Total phosphoprotein** |  |  |  |  |  |  |
| Logistic model^b^ | 305 | Ref. | NA | NA | 0.79 (-1.03 - 2.60) | 0.4 |
| Gamma model^c^ | 298 | Ref. | 10.7% (-17.4% - 50.7%) | 0.5 | 23.4% (-0.5% - 53.5%) | 0.052 |
| **p-mTOR/mTOR** |  |  |  |  |  |  |
| Logistic model^b^ | 307 | Ref. | 1.1 (0.34 - 4.51) | 0.88 | 1.64 (0.66 - 4.38) | 0.3 |
| Gamma model^c^ | 260 | Ref. | 0.8% (-29.6% - 47.5%) | 0.97 | -0.5% (-23.6% - 30.2%) | 0.97 |

^a^All models adjusted for the same covariates except for the stratified variable.

^b^The first part of the gamma hurdle model, i.e., modeling positive (H-score >0) vs. negative (H-score =0) expression with a logistic model.

^c^The second part of the gamma hurdle model, i.e., modeling the positive expression (H-score >0) with a gamma model.

Abbreviations: CI, confidence interval; NA, not applicable; Ref., reference.

1. **High grade**

|  |  | Physical activity levels | | | | |
| --- | --- | --- | --- | --- | --- | --- |
| Protein expression (Outcome)^a^ | No. | No | Insufficient |  | Sufficient |  |
|  |  |  | Difference or odds ratio (95% CI) | P value | Difference or odds ratio (95% CI) | P value |
| **mTOR** |  |  |  |  |  |  |
| Linear model | 287 | Ref. | -2.52 (-29.08 - 24.04) | 0.85 | 10.24 (-9.09 - 29.57) | 0.3 |
| **p-mTOR** |  |  |  |  |  |  |
| Logistic model^b^ | 282 | Ref. | 1.99 (0.65 - 7.6) | 0.26 | 1.58 (0.7 - 3.76) | 0.28 |
| Gamma model^c^ | 243 | Ref. | 22.7% (-32.1% - 131.7%) | 0.48 | -1.4% (-36.5% - 54.2%) | 0.95 |
| **p-AKT** |  |  |  |  |  |  |
| Logistic model^b^ | 284 | Ref. | 0.77 (0.34 - 1.79) | 0.012 | 1.06 (0.57 - 1.98) | 0.078 |
| Gamma model^c^ | 181 | Ref. | NA | NA | NA | NA |
| **p-P70S6K** |  |  |  |  |  |  |
| Logistic model^b^ | 282 | Ref. | 0.85 (0.34 - 2.3) | 0.74 | 1.61 (0.77 - 3.52) | 0.21 |
| Gamma model^c^ | 227 | Ref. | 12.6% (-40.3% - 121.1%) | 0.69 | 40.6% (-10.7% - 121.6%) | 0.12 |
| **Total phosphoprotein** |  |  |  |  |  |  |
| Logistic model^b^ | 280 | Ref. | NA | NA | 1.65 (0.41 - 7.79) | 0.5 |
| Gamma model^c^ | 268 | Ref. | 19.3% (-26.9% - 100.3%) | 0.46 | 34.8% (-7.3% - 97.3%) | 0.096 |
| **p-mTOR/mTOR** |  |  |  |  |  |  |
| Logistic model^b^ | 280 | Ref. | 1.89 (0.61 - 7.22) | 0.3 | 1.81 (0.78 - 4.51) | 0.18 |
| Gamma model^c^ | 226 | Ref. | 9.9% (-37.7% - 103.4%) | 0.74 | -13.3% (-42% - 30.3%) | 0.48 |

^a^All models adjusted for the same covariates except for the stratified variable.

^b^The first part of the gamma hurdle model, i.e., modeling positive (H-score >0) vs. negative (H-score =0) expression with a logistic model.

^c^The second part of the gamma hurdle model, i.e., modeling the positive expression (H-score >0) with a gamma model.

Abbreviations: CI, confidence interval; NA, not applicable; Ref., reference.
