# Supplementary material for: Association Between Recreational Physical Activity and mTOR Signaling Pathway Protein Expression in Breast Tumor Tissue
Source: Cancer Res Commun. 2023 Mar 7;3(3):395–403. doi: 10.1158/2767-9764.CRC-22-0405 (PMC9990525; doi:10.1158/2767-9764.CRC-22-0405)
Supplement: Supplemental Table 13 — reported stratified analysis for tumors <2cm and ≥2 cm in diameter. [file crc-22-0405-s13.docx]

Supplemental Table 13. Stratified analysis by tumor size

1. **Tumor size <2 cm in diameter**

|  |  | Physical activity levels | | | | |
| --- | --- | --- | --- | --- | --- | --- |
| Protein expression (Outcome)^a^ | No. | No | Insufficient |  | Sufficient |  |
|  |  |  | Difference or odds ratio (95% CI) | P value | Difference or odds ratio (95% CI) | P value |
| **mTOR** |  |  |  |  |  |  |
| Linear model | 306 | Ref. | 7.17 (-17.86 - 32.21) | 0.57 | 13.35 (-4.67 - 31.37) | 0.15 |
| **p-mTOR** |  |  |  |  |  |  |
| Logistic model^b^ | 300 | Ref. | 1.49 (0.47 - 5.83) | 0.53 | 1.49 (0.61 - 3.85) | 0.39 |
| Gamma model^c^ | 268 | Ref. | 59.5% (4.8% - 150.1%) | 0.029 | 16.5% (-13% - 56.6%) | 0.3 |
| **p-AKT** |  |  |  |  |  |  |
| Logistic model^b^ | 305 | Ref. | 1.71 (0.72 - 4.45) | 0.24 | 1.5 (0.81 - 2.83) | 0.21 |
| Gamma model^c^ | 230 | Ref. | -0.5% (-38.2% - 65.7%) | 0.98 | 1.5% (-28.3% - 44%) | 0.93 |
| **p-P70S6K** |  |  |  |  |  |  |
| Logistic model^b^ | 304 | Ref. | 1.72 (0.72 - 4.47) | 0.24 | 2.05 (1.06 - 4.09) | 0.036 |
| Gamma model^c^ | 235 | Ref. | 18.1% (-27.8% - 100.5%) | 0.51 | 49.8% (4.1% - 116.1%) | 0.026 |
| **Total phosphoprotein** |  |  |  |  |  |  |
| Logistic model^b^ | 298 | Ref. | NA | NA | 2.29 (0.56 - 12.05) | 0.27 |
| Gamma model^c^ | 287 | Ref. | 24.2% (-11.6% - 78.1%) | 0.22 | 26% (-1.3% - 61.2%) | 0.069 |
| **p-mTOR/mTOR** |  |  |  |  |  |  |
| Logistic model^b^ | 298 | Ref. | 1.58 (0.49 - 6.29) | 0.47 | 1.99 (0.77 - 5.59) | 0.17 |
| Gamma model^c^ | 253 | Ref. | 31.4% (-12.5% - 103.6%) | 0.19 | 19.6% (-9.9% - 59.2%) | 0.21 |

^a^All models adjusted for the same covariates except for the stratified variable.

^b^The first part of the gamma hurdle model, i.e., modeling positive (H-score >0) vs. negative (H-score =0) expression with a logistic model.

^c^The second part of the gamma hurdle model, i.e., modeling the positive expression (H-score >0) with a gamma model.

Abbreviations: CI, confidence interval; NA, not applicable; Ref., reference.

1. **Tumor size ≥2 cm in diameter**

|  |  | Physical activity levels | | | | |
| --- | --- | --- | --- | --- | --- | --- |
| Protein expression (Outcome)^a^ | No. | No | Insufficient |  | Sufficient |  |
|  |  |  | Difference or odds ratio (95% CI) | P value | Difference or odds ratio (95% CI) | P value |
| **mTOR** |  |  |  |  |  |  |
| Linear model | 293 | Ref. | -4.15 (-29.86 - 21.56) | 0.75 | 5.29 (-13.92 - 24.49) | 0.59 |
| **p-mTOR** |  |  |  |  |  |  |
| Logistic model^b^ | 293 | Ref. | 1.34 (0.44 - 5.02) | 0.63 | 1.35 (0.6 - 3.17) | 0.47 |
| Gamma model^c^ | 255 | Ref. | -16.9% (-47.4% - 36.4%) | 0.43 | 12.9% (-21.3% - 63.4%) | 0.5 |
| **p-AKT** |  |  |  |  |  |  |
| Logistic model^b^ | 293 | Ref. | 1.55 (0.69 - 3.69) | 0.3 | 1.21 (0.68 - 2.16) | 0.52 |
| Gamma model^c^ | 191 | Ref. | -6.9% (-46.1% - 67.7%) | 0.8 | 12.3% (-27.3% - 75.6%) | 0.59 |
| **p-P70S6K** |  |  |  |  |  |  |
| Logistic model^b^ | 291 | Ref. | 0.95 (0.39 - 2.53) | 0.91 | 1.27 (0.63 - 2.61) | 0.51 |
| Gamma model^c^ | 232 | Ref. | 11.3% (-38.3% - 109.7%) | 0.71 | 26% (-20.5% - 101.7%) | 0.28 |
| **Total phosphoprotein** |  |  |  |  |  |  |
| Logistic model^b^ | 287 | Ref. | NA | NA | 1.54 (0.26 - 12.08) | 0.65 |
| Gamma model^c^ | 279 | Ref. | 11.9% (-24.8% - 71.1%) | 0.58 | 30.1% (-5.4% - 79.9%) | 0.089 |
| **p-mTOR/mTOR** |  |  |  |  |  |  |
| Logistic model^b^ | 289 | Ref. | 1.21 (0.4 - 4.56) | 0.75 | 1.43 (0.62 - 3.47) | 0.41 |
| Gamma model^c^ | 234 | Ref. | -6.3% (-40.9% - 54.4%) | 0.79 | 2.1% (-29% - 48.1%) | 0.91 |

^a^All models adjusted for the same covariates except for the stratified variable.

^b^The first part of the gamma hurdle model, i.e., modeling positive (H-score >0) vs. negative (H-score =0) expression with a logistic model.

^c^The second part of the gamma hurdle model, i.e., modeling the positive expression (H-score >0) with a gamma model.

Abbreviations: CI, confidence interval; NA, not applicable; Ref., reference.
