# Supplementary material for: Association Between Recreational Physical Activity and mTOR Signaling Pathway Protein Expression in Breast Tumor Tissue
Source: Cancer Res Commun. 2023 Mar 7;3(3):395–403. doi: 10.1158/2767-9764.CRC-22-0405 (PMC9990525; doi:10.1158/2767-9764.CRC-22-0405)
Supplement: Supplemental Table 14 — reported stratified analysis for ER+ tumors and ER- tumors. [file crc-22-0405-s14.docx]

Supplemental Table 14. Stratified analysis by ER status

1. **ER+ tumors**

|  |  | Physical activity levels | | | | |
| --- | --- | --- | --- | --- | --- | --- |
| Protein expression (Outcome)^a^ | No. | No | Insufficient |  | Sufficient |  |
|  |  |  | Difference or odds ratio (95% CI) | P value | Difference or odds ratio (95% CI) | P value |
| **mTOR** |  |  |  |  |  |  |
| Linear model | 441 | Ref. | 2.16 (-17.85 - 22.18) | 0.83 | 9.92 (-5.44 - 25.28) | 0.2 |
| **p-mTOR** |  |  |  |  |  |  |
| Logistic model^b^ | 439 | Ref. | 1.06 (0.41 - 3.13) | 0.9 | 1.26 (0.58 - 2.84) | 0.56 |
| Gamma model^c^ | 399 | Ref. | 16.1% (-12.9% - 57.1%) | 0.31 | 17.8% (-5.6% - 47.3%) | 0.15 |
| **p-AKT** |  |  |  |  |  |  |
| Logistic model^b^ | 440 | Ref. | 1.78 (0.89 - 3.77) | 0.12 | 1.2 (0.73 - 2) | 0.47 |
| Gamma model^c^ | 325 | Ref. | 21% (-16.1% - 78%) | 0.32 | 38.9% (3.8% - 86.9%) | 0.025 |
| **p-P70S6K** |  |  |  |  |  |  |
| Logistic model^b^ | 440 | Ref. | 1.63 (0.78 - 3.7) | 0.21 | 1.35 (0.77 - 2.39) | 0.3 |
| Gamma model^c^ | 353 | Ref. | 16.3% (-21% - 75%) | 0.44 | 36.5% (0.7% - 85.8%) | 0.041 |
| **Total phosphoprotein** |  |  |  |  |  |  |
| Logistic model^b^ | 431 | Ref. | NA | NA | 1.3 (0.37 - 5.34) | 0.69 |
| Gamma model^c^ | 419 | Ref. | 28.8% (0.3% - 67.1%) | 0.049 | 35.9% (11.9% - 65.5%) | 0.0019 |
| **p-mTOR/mTOR** |  |  |  |  |  |  |
| Logistic model^b^ | 432 | Ref. | 0.96 (0.36 - 2.85) | 0.93 | 1.53 (0.67 - 3.75) | 0.33 |
| Gamma model^c^ | 371 | Ref. | 12.7% (-15.9% - 53.5%) | 0.43 | -1.8% (-21.6% - 23.3%) | 0.87 |

^a^All models adjusted for the same covariates except for the stratified variable.

^b^The first part of the gamma hurdle model, i.e., modeling positive (H-score >0) vs. negative (H-score =0) expression with a logistic model.

^c^The second part of the gamma hurdle model, i.e., modeling the positive expression (H-score >0) with a gamma model.

Abbreviations: CI, confidence interval; NA, not applicable; Ref., reference.

1. **ER- tumors**

|  |  | Physical activity levels | | | | |
| --- | --- | --- | --- | --- | --- | --- |
| Protein expression (Outcome)^a^ | No. | No | Insufficient |  | Sufficient |  |
|  |  |  | Difference or odds ratio (95% CI) | P value | Difference or odds ratio (95% CI) | P value |
| **mTOR** |  |  |  |  |  |  |
| Linear model | 170 | Ref. | -13.34 (-51.09 - 24.42) | 0.49 | 7.76 (-16.34 - 31.86) | 0.53 |
| **p-mTOR** |  |  |  |  |  |  |
| Logistic model^b^ | 167 | Ref. | 2.52 (0.59 - 17.66) | 0.26 | 1.66 (0.66 - 4.44) | 0.29 |
| Gamma model^c^ | 136 | Ref. | -34.2% (-75.5% - 100.5%) | 0.37 | -11.3% (-53.3% - 71%) | 0.69 |
| **p-AKT** |  |  |  |  |  |  |
| Logistic model^b^ | 172 | Ref. | 0.87 (0.28 - 2.74) | 0.81 | 1.81 (0.85 - 3.97) | 0.13 |
| Gamma model^c^ | 106 | Ref. | -14% (-66.8% - 163.3%) | 0.76 | -31.3% (-65.8% - 39%) | 0.25 |
| **p-P70S6K** |  |  |  |  |  |  |
| Logistic model^b^ | 168 | Ref. | 0.66 (0.21 - 2.16) | 0.48 | 1.99 (0.85 - 4.95) | 0.12 |
| Gamma model^c^ | 123 | Ref. | -5.5% (-41.6% - 229.9%) | 0.91 | 25.5% (-35% - 51%) | 0.45 |
| **Total phosphoprotein** |  |  |  |  |  |  |
| Logistic model^b^ | 167 | Ref. | NA | NA | 2.95 (0.45 - 31.86) | 0.3 |
| Gamma model^c^ | 160 | Ref. | -10.7% (-56.8% - 98.3%) | 0.74 | 10% (-31.8% - 79%) | 0.67 |
| **p-mTOR/mTOR** |  |  |  |  |  |  |
| Logistic model^b^ | 167 | Ref. | 2.52 (0.59 - 17.66) | 0.26 | 1.66 (0.66 - 4.44) | 0.29 |
| Gamma model^c^ | 118 | Ref. | -5.8% (-61.5% - 155.6%) | 0.89 | -4.7% (-47.6% - 76.2%) | 0.87 |

^a^All models adjusted for the same covariates except for the stratified variable.

^b^The first part of the gamma hurdle model, i.e., modeling positive (H-score >0) vs. negative (H-score =0) expression with a logistic model.

^c^The second part of the gamma hurdle model, i.e., modeling the positive expression (H-score >0) with a gamma model.

Abbreviations: CI, confidence interval; NA, not applicable; Ref., reference.
