# Supplementary material for: Association Between Recreational Physical Activity and mTOR Signaling Pathway Protein Expression in Breast Tumor Tissue
Source: Cancer Res Commun. 2023 Mar 7;3(3):395–403. doi: 10.1158/2767-9764.CRC-22-0405 (PMC9990525; doi:10.1158/2767-9764.CRC-22-0405)
Supplement: Supplemental Table 15 — reported stratified analysis for lymph node positive status and lymph node negative status. [file crc-22-0405-s15.docx]

Supplemental Table 15. Stratified analysis by lymph node status

1. **Lymph node positive**

|  |  | Physical activity levels | | | | |
| --- | --- | --- | --- | --- | --- | --- |
| Protein expression (Outcome)^a^ | No. | No | Insufficient |  | Sufficient |  |
|  |  |  | Difference or odds ratio (95% CI) | P value | Difference or odds ratio (95% CI) | P value |
| **mTOR** |  |  |  |  |  |  |
| Linear model | 226 | Ref. | -13.1 (-42.88 - 16.67) | 0.39 | -11.75 (-32.82 - 9.33) | 0.27 |
| **p-mTOR** |  |  |  |  |  |  |
| Logistic model^b^ | 226 | Ref. | 0.81 (0.21 - 4.04) | 0.77 | 0.49 (0.19 - 1.27) | 0.14 |
| Gamma model^c^ | 198 | Ref. | 14.8% (-35.7% - 117.8%) | 0.64 | 24.5% (-18.5% - 92.7%) | 0.31 |
| **p-AKT** |  |  |  |  |  |  |
| Logistic model^b^ | 225 | Ref. | 1.96 (0.72 - 5.8) | 0.2 | 1.29 (0.66 - 2.56) | 0.46 |
| Gamma model^c^ | 143 | Ref. | 4.5% (-40.8% - 94.2%) | 0.88 | 23.4% (-24.1% - 103.1%) | 0.35 |
| **p-P70S6K** |  |  |  |  |  |  |
| Logistic model^b^ | 225 | Ref. | 3.63 (1.07 - 16.96) | 0.06 | 1.57 (0.73 - 3.53) | 0.26 |
| Gamma model^c^ | 174 | Ref. | -8.2% (-51.7% - 83%) | 0.78 | -7.3% (-43.2% - 52.6%) | 0.75 |
| **Total phosphoprotein** |  |  |  |  |  |  |
| Logistic model^b^ | 222 | Ref. | NA | NA | 0.71 (0.12 - 4.67) | 0.71 |
| Gamma model^c^ | 214 | Ref. | 23.4% (-21.5% - 101.2%) | 0.37 | 19.8% (-14.6% - 69.2%) | 0.28 |
| **p-mTOR/mTOR** |  |  |  |  |  |  |
| Logistic model^b^ | 223 | Ref. | 0.91 (0.23 - 4.66) | 0.9 | 0.56 (0.21 - 1.53) | 0.26 |
| Gamma model^c^ | 182 | Ref. | 10.7% (-35.2% - 100.3%) | 0.72 | 9.1% (-27.7% - 66.5%) | 0.67 |

^a^All models adjusted for the same covariates except for the stratified variable.

^b^The first part of the gamma hurdle model, i.e., modeling positive (H-score >0) vs. negative (H-score =0) expression with a logistic model.

^c^The second part of the gamma hurdle model, i.e., modeling the positive expression (H-score >0) with a gamma model.

Abbreviations: CI, confidence interval; NA, not applicable; Ref., reference.

1. **Lymph node negative**

|  |  | Physical activity levels | | | | |
| --- | --- | --- | --- | --- | --- | --- |
| Protein expression (Outcome)^a^ | No. | No | Insufficient |  | Sufficient |  |
|  |  |  | Difference or odds ratio (95% CI) | P value | Difference or odds ratio (95% CI) | P value |
| **mTOR** |  |  |  |  |  |  |
| Linear model | 309 | Ref. | 5.06 (-19.4 - 29.52) | 0.68 | 14.68 (-3.08 - 32.45) | 0.1 |
| **p-mTOR** |  |  |  |  |  |  |
| Logistic model^b^ | 304 | Ref. | 1.97 (0.64 - 7.64) | 0.27 | 3.47 (1.42 - 9.55) | 0.0097 |
| Gamma model^c^ | 268 | Ref. | 34.8% (-13.5% - 116.1%) | 0.17 | 15.5% (-16.7% - 60.5%) | 0.36 |
| **p-AKT** |  |  |  |  |  |  |
| Logistic model^b^ | 309 | Ref. | 1.87 (0.8 - 4.8) | 0.16 | 1.42 (0.78 - 2.6) | 0.26 |
| Gamma model^c^ | 229 | Ref. | 7.9% (-34.6% - 83.9%) | 0.77 | 18.1% (-20.1% - 75.5%) | 0.39 |
| **p-P70S6K** |  |  |  |  |  |  |
| Logistic model^b^ | 306 | Ref. | 0.92 (0.4 - 2.27) | 0.86 | 1.4 (0.71 - 2.85) | 0.34 |
| Gamma model^c^ | 243 | Ref. | 31.8% (-24.2% - 139.2%) | 0.32 | 70.8% (11.1% - 163.3%) | 0.0084 |
| **Total phosphoprotein** |  |  |  |  |  |  |
| Logistic model^b^ | 300 | Ref. | NA | NA | 2.08 (0.39 - 16.12) | 0.42 |
| Gamma model^c^ | 292 | Ref. | 29% (-10% - 88.4%) | 0.16 | 40.7% (7.1% - 85.3%) | 0.011 |
| **p-mTOR/mTOR** |  |  |  |  |  |  |
| Logistic model^b^ | 301 | Ref. | 1.98 (0.63 - 7.69) | 0.28 | 3.86 (1.51 - 11.4) | 0.0079 |
| Gamma model^c^ | 248 | Ref. | 26.1% (-17.1% - 97.1%) | 0.29 | 6.1% (-23.2% - 47.2%) | 0.71 |

^a^All models adjusted for the same covariates except for the stratified variable.

^b^The first part of the gamma hurdle model, i.e., modeling positive (H-score >0) vs. negative (H-score =0) expression with a logistic model.

^c^The second part of the gamma hurdle model, i.e., modeling the positive expression (H-score >0) with a gamma model.

Abbreviations: CI, confidence interval; NA, not applicable; Ref., reference.
