# Supplementary material for: Association Between Recreational Physical Activity and mTOR Signaling Pathway Protein Expression in Breast Tumor Tissue
Source: Cancer Res Commun. 2023 Mar 7;3(3):395–403. doi: 10.1158/2767-9764.CRC-22-0405 (PMC9990525; doi:10.1158/2767-9764.CRC-22-0405)
Supplement: Supplemental Table 16 — reported stratified analysis for high (above the median) and low (at or below the median) total energy intake levels. [file crc-22-0405-s16.docx]

Supplemental Table 16. Stratified analysis by the median level of total energy intake

1. **Total energy intake level above the median**

|  |  | Physical activity levels | | | | | |
| --- | --- | --- | --- | --- | --- | --- | --- |
| Protein expression (Outcome)^a^ | No. | No | Insufficient |  | Sufficient |  |  |
|  |  |  | Difference or odds ratio (95% CI) | P value | Difference or odds ratio (95% CI) | P value |  |
| **mTOR** |  |  |  |  |  |  |  |
| Linear model | 295 | Ref. | -7.84 (-32.25 - 16.57) | 0.53 | 15.38 (-2.71 - 33.47) | 0.095 |  |
| **p-mTOR** |  |  |  |  |  |  |  |
| Logistic model^b^ | 296 | Ref. | 1.61 (0.56 - 5.48) | 0.4 | 2.04 (0.89 - 4.93) | 0.099 |  |
| Gamma model^c^ | 259 | Ref. | 26.3% (-17.7% - 99.5%) | 0.29 | 29.7% (-6.8% - 81.2%) | 0.11 |  |
| **p-AKT** |  |  |  |  |  |  |  |
| Logistic model^b^ | 297 | Ref. | 1.81 (0.78 - 4.52) | 0.18 | 1.56 (0.85 - 2.93) | 0.16 |  |
| Gamma model^c^ | 216 | Ref. | 11.1% (-31.4% - 85.4%) | 0.67 | 7.4% (-26.7% - 58.1%) | 0.7 |  |
| **p-P70S6K** |  |  |  |  |  |  |  |
| Logistic model^b^ | 296 | Ref. | 0.96 (0.41 - 2.42) | 0.93 | 1.48 (0.73 - 3.11) | 0.29 |  |
| Gamma model^c^ | 244 | Ref. | 25.9% (-22.3% - 103.7%) | 0.35 | 44.4% (1% - 106.6%) | 0.045 |  |
| **Total phosphoprotein** |  |  |  |  |  |  |  |
| Logistic model^b^ | 290 | Ref. | NA | NA | 4.2 (0.66 - 51.67) | 0.18 |  |
| Gamma model^c^ | 282 | Ref. | 27.1% (-10.9% - 84.5%) | 0.18 | 31.3% (0.5% - 71.8%) | 0.042 |  |
| **p-mTOR/mTOR** |  |  |  |  |  |  |  |
| Logistic model^b^ | 291 | Ref. | 1.56 (0.53 - 5.34) | 0.45 | 2.31 (0.97 - 5.98) | 0.068 |  |
| Gamma model^c^ | 242 | Ref. | 40.4% (-7.7% - 119.9%) | 0.12 | 17% (-14.1% - 59.8%) | 0.32 |  |

^a^All models adjusted for the same covariates except for the stratified variable.

^b^The first part of the gamma hurdle model, i.e., modeling positive (H-score >0) vs. negative (H-score =0) expression with a logistic model.

^c^The second part of the gamma hurdle model, i.e., modeling the positive expression (H-score >0) with a gamma model.

Abbreviations: CI, confidence interval; NA, not applicable; Ref., reference.

1. **Total energy intake level at or below the median**

|  |  | Physical activity levels | | | | | |
| --- | --- | --- | --- | --- | --- | --- | --- |
| Protein expression (Outcome)^a^ | No. | No | Insufficient |  | Sufficient |  |  |
|  |  |  | Difference or odds ratio (95% CI) | P value | Difference or odds ratio (95% CI) | P value |  |
| **mTOR** |  |  |  |  |  |  |  |
| Linear model | 294 | Ref. | 7.87 (-19.13 - 34.88) | 0.57 | -3.57 (-22.79 - 15.65) | 0.71 |  |
| **p-mTOR** |  |  |  |  |  |  |  |
| Logistic model^b^ | 287 | Ref. | 2.35 (0.59 - 15.89) | 0.29 | 1.19 (0.49 - 2.97) | 0.71 |  |
| Gamma model^c^ | 256 | Ref. | -23.9% (-53.4% - 29.4%) | 0.27 | -16% (-40.6% - 19.9%) | 0.33 |  |
| **p-AKT** |  |  |  |  |  |  |  |
| Logistic model^b^ | 291 | Ref. | 1.24 (0.53 - 3.09) | 0.63 | 1.2 (0.66 - 2.21) | 0.56 |  |
| Gamma model^c^ | 200 | Ref. | 6.5% (-40.4% - 100.9%) | 0.83 | 32.2% (-16.5% - 110.3%) | 0.2 |  |
| **p-P70S6K** |  |  |  |  |  |  |  |
| Logistic model^b^ | 289 | Ref. | 1.97 (0.76 - 5.82) | 0.18 | 1.57 (0.81 - 3.1) | 0.19 |  |
| Gamma model^c^ | 217 | Ref. | -6.5% (-51.2% - 88.5%) | 0.83 | 26% (-22.4% - 105.4%) | 0.32 |  |
| **Total phosphoprotein** |  |  |  |  |  |  |  |
| Logistic model^b^ | 285 | Ref. | NA | NA | 0.94 (0.21 - 4.58) | 0.94 |  |
| Gamma model^c^ | 275 | Ref. | 2.8% (-31.7% - 59.6%) | 0.89 | 21.5% (-10.5% - 65.8%) | 0.2 |  |
| **p-mTOR/mTOR** |  |  |  |  |  |  |  |
| Logistic model^b^ | 286 | Ref. | 2.28 (0.57 - 15.47) | 0.3 | 1.29 (0.53 - 3.34) | 0.58 |  |
| Gamma model^c^ | 236 | Ref. | -29.2% (-55.7% - 18.3%) | 0.16 | -11.6% (-37.8% - 26.5%) | 0.49 |  |

^a^All models adjusted for the same covariates except for the stratified variable.

^b^The first part of the gamma hurdle model, i.e., modeling positive (H-score >0) vs. negative (H-score =0) expression with a logistic model.

^c^The second part of the gamma hurdle model, i.e., modeling the positive expression (H-score >0) with a gamma model.

Abbreviations: CI, confidence interval; NA, not applicable; Ref., reference.
